# Unconventional bipartite entanglement in the quantum dimer magnet Yb_2_Be_2_SiO_7_

**DOI:** 10.1038/s41467-026-69258-7

**Published:** 2026-03-25

**Authors:** A. Brassington, Q. Ma, G. Duan, S. Calder, A. I. Kolesnikov, K. M. Taddei, G. Sala, E. S. Choi, H. Wang, W. Xie, B. A. Frandsen, N. Li, X. F. Sun, C. Liu, R. Yu, H. D. Zhou, A. A. Aczel

**Affiliations:** 1 Department of Physics and Astronomy, University of Tennessee, Knoxville, TN USA; 2 Neutron Scattering Division, Oak Ridge National Laboratory, Oak Ridge, TN USA; 3 School of Physics and Beijing Key Laboratory of Optoelectronic Functional Materials and Micro-nano Devices, Renmin University of China, Beijing, PR China; 4 Oak Ridge National Laboratory, Oak Ridge, TN USA; 5 National High Magnetic Field Laboratory and Department of Physics, Florida State University, Tallahassee, FL USA; 6 Department of Chemistry, Michigan State University, East Lansing, MI USA; 7 Department of Physics and Astronomy, Brigham Young University, Provo, UT USA; 8 Anhui Provincial Key Laboratory of Magnetic Functional Materials and Devices, Institutes of Physical Science and Information Technology, Anhui University, Hefei, Anhui PR China; 9 School of Engineering, Dali University, Dali, Yunnan PR China; 10 Key Laboratory of Quantum State Construction and Manipulation (Ministry of Education), Renmin University of China, Beijing, PR China; 11 Present Address: X-ray Sciences Division, Advanced Photon Source, Argonne National Laboratory, Lemont, Illinois USA; 12 Present Address: J-PARC Center, Japan Atomic Energy Agency (JAEA), Materials and Life Science, Ibaraki, Japan; 13 Present Address: School of Physics and Mechatronic Engineering, Guizhou Minzu University, Guiyang, Guizhou PR China

**Keywords:** Magnetic properties and materials, Magnetic properties and materials

## Abstract

The quantum dimer magnet, with antiferromagnetic intradimer and interdimer Heisenberg exchange between spin-1/2 moments, is known to host an $(\left|\uparrow \downarrow \right\rangle -\left|\downarrow \uparrow \right\rangle )/\sqrt{2}$ singlet ground state when the intradimer exchange is dominant. Rare-earth-based quantum dimer systems with strong spin-orbit coupling offer the opportunity for tuning their magnetic properties by using magnetic anisotropy as a control knob. Here, we present bulk characterization and neutron scattering measurements of the quantum dimer magnet Yb_2_Be_2_SiO_7_. We find that the Yb^3+^ ions can be described by an effective spin-1/2 model at low temperatures and the system does not show signs of magnetic order down to 50 mK. The magnetization, heat capacity, and neutron spectroscopy data can be well-described by an isolated dimer model with highly anisotropic exchange that stabilizes a singlet ground state with a wavefunction $(\left|\uparrow \uparrow \right\rangle -\left|\downarrow \downarrow \right\rangle )/\sqrt{2}$ or $(\left|\uparrow \uparrow \right\rangle+\left|\downarrow \downarrow \right\rangle )/\sqrt{2}$. Our results show that strong spin-orbit coupling can induce unusual entangled states of matter in quantum dimer magnets.

## Abstract (web summary)

The Shastry-Sutherland model consists of orthogonal dimers in a two dimensional plane, and has proved a rich basis for both theoretical and experimental investigation of quantum magnetism. Here, Brassington et al show that Yb2Be2SiO7 hosts an anisotropic variant of the Shastry Sutherland model.

## Introduction

Insulating magnets devoid of ground states with long-range order and instead characterized by strongly-interacting units featuring quantum entanglement^1,2^ have attracted intense interest in the field of quantum magnetism. The simplest case consists of a series of dimers with two entangled spins per unit^3,4^, which is known as a quantum dimer magnet for spin-1/2. The Shastry-Sutherland lattice (SSL) consists of a plane of orthogonal dimers^5,6^, and it is now known as one of the canonical two-dimensional models that can host entangled spin states. For isotropic Heisenberg spins on the SSL with a sufficiently small ratio of interdimer-to-intradimer exchange interactions *α* = *J*_2_/*J*_1_, the Hamiltonian is exactly solvable and the ground state is predicted to be a direct product of entangled spin-singlet states. The gapped elementary excitations associated with this exotic state are known as triplons^7,8^.

SSL research progressed beyond the initial theoretical studies of the simple toy model with the discovery of SrCu_2_(BO_3_)_2_^9,10^, which consists of weakly-coupled planes of $S=\frac{1}{2}$ Cu^2+^ ions with the desired orthogonal dimer geometry^11^. The *α* ratio is sufficiently small to generate a spin-singlet ground state^9,10^, although it is close to the critical value required to produce Neel antiferromagnetic order^11,12^. This proximity to a quantum phase transition has now been investigated in great detail by both experiment and theory, with a pressure-induced singlet plaquette phase first identified by neutron spectroscopy^13^ and multiple theoretical studies predicting quantum spin liquid phases in this regime as well^14–17^. SrCu_2_(BO_3_)_2_ also exhibits striking magnetic-field-induced phenomena, with several magnetization plateaus observed that were first explained by triplon crystallization^18–20^. The lower-field plateaus were later argued to arise from triplon bound state crystallization instead^21^.

More recently, the effect of strong spin-orbit-coupling on the magnetic properties of the SSL has received significant attention. Relevant theoretical work has explored the magnetic properties of the Ising or XXZ SSL Hamiltonian^22–27^, which can be realized for systems with significant magnetic anisotropy. On the experimental side, the *R*B_4_ (*R* = rare earth) family members were initially explored as possible anisotropic SSL systems^28–33^. Unfortunately, they are itinerant magnets with long-range Ruderman–Kittel–Kasuya–Yosida (RKKY) exchange interactions mediated by the conduction electrons^34–38^, so their magnetic Hamiltonians are more complex than the simple *J*_1_-*J*_2_ (or nearest-neighbor and next-nearest-neighbor) SSL model. Two insulating families Ba*R*_2_M*X*_5_ (*M* = Zn, Pd, or Pt; *X* = O or S)^39–46^ and *R*_2_Be_2_*Z*O_7_ (*Z* = Ge or Si)^47,48^ have now come to the forefront as superior model systems for exploring SSL physics in strongly anisotropic magnets. A subset of these rare-earth-based materials host effective spin-1/2 degrees of freedom at sufficiently low temperatures, which arise from a well-isolated crystal field ground state doublet. While ordered ground states have often been identified^40,41,43,45,49–51^, effective spin-1/2 systems can realize more exotic behavior. Both BaCe_2_ZnS_5_^46^ and Yb_2_Be_2_GeO_7_^52,53^ are effective spin-1/2 dimer systems with entangled ground states and the latter has emerged as a quantum spin liquid candidate^52^.

In this work, we report comprehensive bulk characterization and neutron scattering measurements of the quantum dimer magnet Yb_2_Be_2_SiO_7_ with SSL geometry. We find that the Yb^3+^ ions have effective spin-1/2 degrees of freedom at sufficiently low temperatures below 10 K, and we observe no evidence for magnetic order down to dilution-refrigerator temperatures of 50 mK. The strong magnetic anisotropy of this system generates nearly Ising moments with a strong tendency to align along the [001]-axis. The zero-field neutron spectroscopy and the magnetic-field and temperature-dependence of both the magnetization and specific heat data closely match expectations for a novel entangled dimer state. More specifically, Yb_2_Be_2_SiO_7_ consists of a series of dimers with *S*_*z*_ ≠ 0 ground state wavefunctions $(\left|\uparrow \uparrow \right\rangle -\left|\downarrow \downarrow \right\rangle )/\sqrt{2}$ or $(\left|\uparrow \uparrow \right\rangle+\left|\downarrow \downarrow \right\rangle )/\sqrt{2}$. The anisotropic intradimer exchange induced by the strong spin-orbit coupling in this system is responsible for stabilizing this exotic state that cannot be realized by the Heisenberg SSL model for spin-1/2 systems.

## Results

### Crystal structure magnetic susceptibility, and neutron powder diffraction

**Fig. 1:**
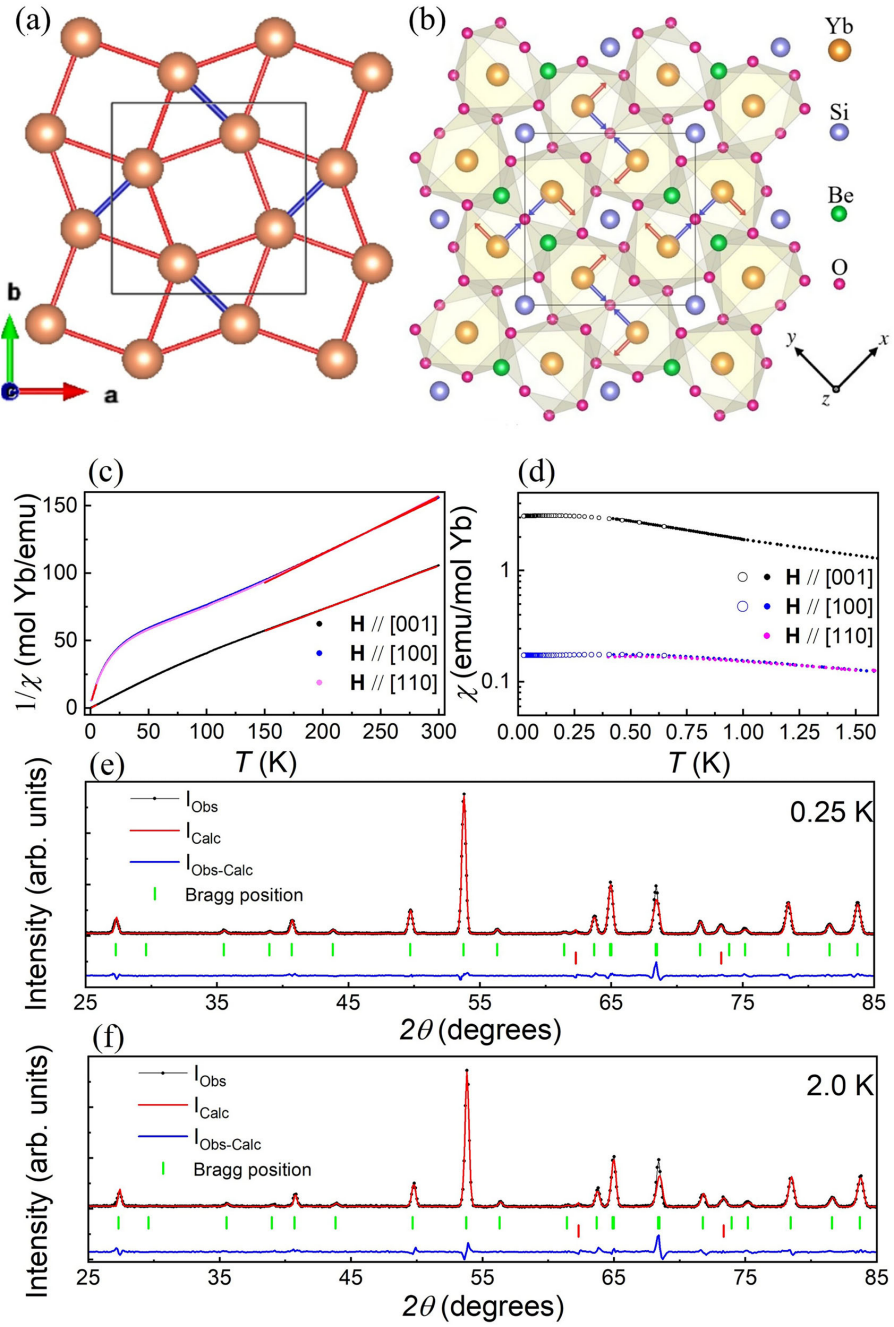
Crystal structure, magnetic susceptibility, and neutron powder diffraction. **a** The arrangement of Yb^3+^ ions in Yb_2_Be_2_SiO_7_ forming an SSL as viewed along the crystallographic [001]-axis. The intradimer and interdimer bonds *J*_1_ and *J*_2_ are shown in blue and red, respectively. **b** The full crystal structure of Yb_2_Be_2_SiO_7_ viewed along the same axis. The four Yb^3+^ ions in the chemical unit cell have g-tensors with different local axes, as explained in the main text. **c** The inverse susceptibility measured in a 0.1 T field applied along three high-symmetry crystallographic directions. The Curie–Weiss fits in both the high and low-temperature regimes are shown in red. **d** The low-*T* susceptibility data plotted along three high-symmetry crystallographic directions. Closed circles and open circles represent DC and AC susceptibility data, respectively. **e**, **f** Neutron powder diffraction patterns collected at 0.25 K and 2 K, respectively. Multi-phase Rietveld refinements with the known Yb_2_Be_2_SiO_7_ crystal structure (green ticks) and Al from the sample can (red ticks) explain the data well. No additional peaks associated with long-range order are observed.

Yb_2_Be_2_SiO_7_ crystallizes into the tetragonal space group *P-42*_1_*m* (113) with room temperature lattice constants *a* = 7.207(1) Å and *c* = 4.719(1) Å determined from single crystal x-ray diffraction (see Supplementary Discussion Section I and Supplementary Tables S1, S2 for more details). The eight-fold coordinated Yb^3+^ ions form an SSL lattice in the *ab*-plane with intradimer and interdimer distances of 3.239(1) Å and 3.836(1) Å, respectively, as shown in Fig. 1a. Adjacent Yb^3+^ planes are separated by 4.719(1) Å. A view of the crystal structure along the *c*-axis with all the atoms shown is depicted in Fig. 1b.

The inverse magnetic susceptibility of single crystalline Yb_2_Be_2_SiO_7_, plotted as field/magnetization *H*/*M*, is presented in Fig. 1c between 0.4 K and 300 K. The data was collected in a small magnetic field of 0.1 T applied along the three high-symmetry directions [001], [110], and [100]. The same data is shown in the low-*T* range between 0.4 K and 1.6 K in Fig. 1d, with the real component of the AC susceptibility data from 0.05 K to 0.65 K also included for two field orientations. There are no clear signatures of long-range magnetic order in this data. Curie–Weiss fits were performed over both high-*T* (150–300 K) and low-*T* (1–5 K) ranges. The effective moment values from the high-*T* fits are broadly consistent with expectations for free Yb^3+^ ions. The low-*T* fits return effective moments and Curie–Weiss temperatures of 4.22(5) *μ*_*B*_ and −0.10(2) K for **H**∥[001], 1.71(1) *μ*_*B*_ and −1.32(1) K for **H**∥[110], and 1.67(1) *μ*_*B*_ and −1.21(1) K for **H**∥[100]. The comparatively large effective moment for **H**∥[001] indicates that the moments have a strong preference to align with the crystallographic [001]-axis at low-*T* and the larger in-plane Curie–Weiss temperatures are consistent with the expected quasi-2D behavior of Yb_2_Be_2_SiO_7_. The small negative Curie–Weiss temperatures are indicative of weak antiferromagnetic interactions in the system under the assumption of isotropic exchange, which is not valid for this system as shown below.

Unpolarized neutron powder diffraction (NPD) patterns collected at 0.25 K and 2 K are presented in Fig. 1e and f, respectively. Both patterns are well-described by nuclear Bragg peaks associated with the Yb_2_Be_2_SiO_7_ crystal structure and the Al sample can. The expected positions of these peaks are indicated by the green and red ticks, respectively. While Rietveld refinements of the data using FULLPROF^54^ reveal no evidence for oxygen off-stoichiometry in this sample, they do identify ~4.4% Be/Si site mixing. Most importantly, there are no additional Bragg peaks or enhanced peak intensities that can be attributed to long-range magnetic order in Yb_2_Be_2_SiO_7_, even down to 0.25 K. Additional details of the 0.25 K refinement are presented in Supplementary Table S3.

### Yb^3+^ single-ion ground state

**Fig. 2:**
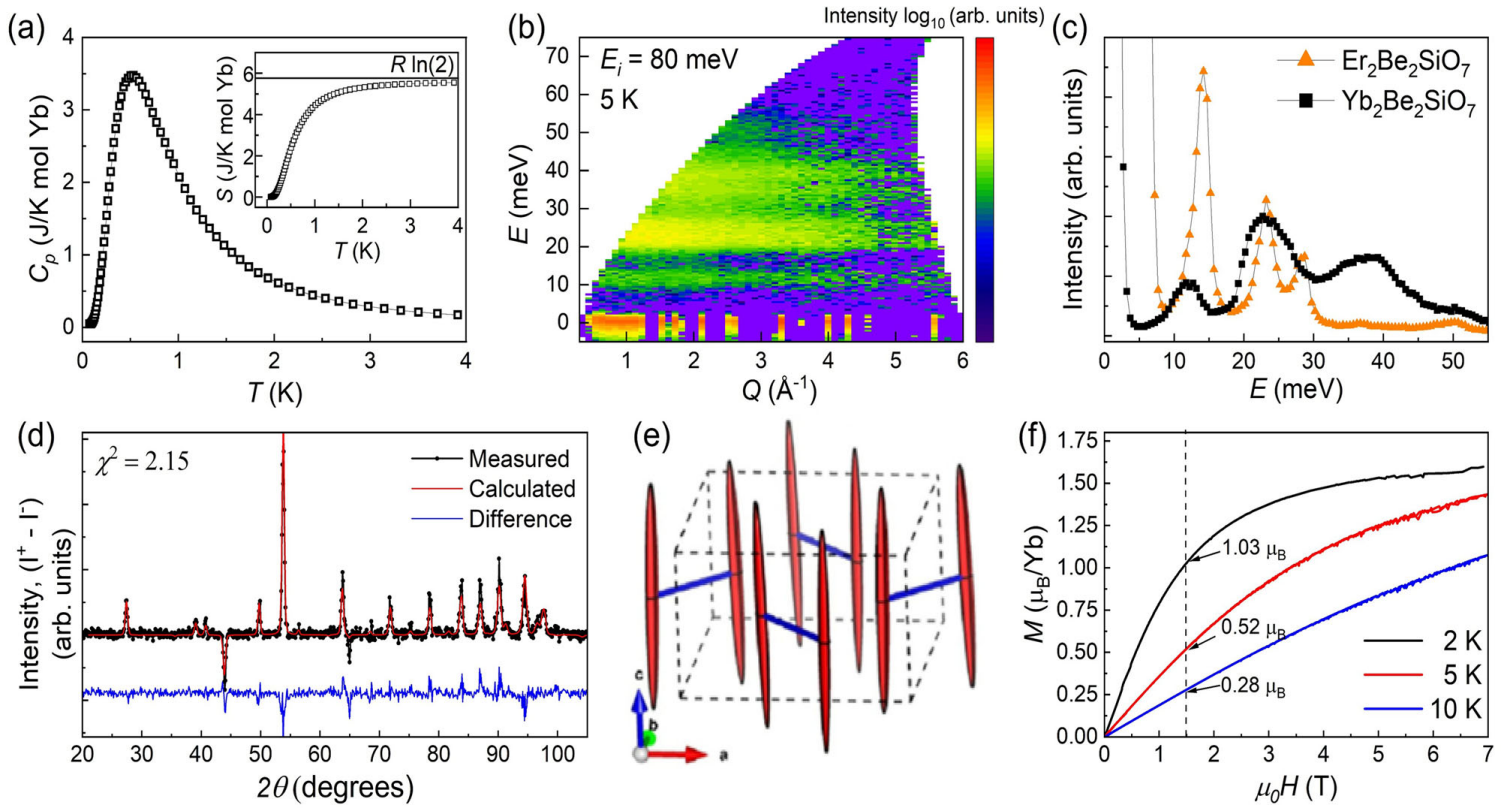
Yb^3+^ single ion ground state. **a** Zero-field heat capacity data reveals a broad Schottky anomaly centered near 0.5 K. The inset shows the magnetic entropy over the same temperature range, which approaches *R*ln(2) at 4 K. **b** Color contour plot of the scattering intensity for the Yb^3+^ ions in Yb_2_Be_2_SiO_7_ as a function of momentum and energy transfer at 5 K from SEQUOIA with *E*_*i*_ = 80 meV. This difference map was obtained by subtracting scaled Lu_2_Be_2_SiO_7_ data to account for the variation in the Yb and Lu neutron scattering cross sections. **c** Constant-*Q* cuts (*Q* integration range [0.25, 5] Å^−1^) of the same Yb_2_Be_2_SiO_7_ data (with no Lu subtraction) and the equivalent data for the magnetic Er^51^ analog. **d** The difference pattern, I^+^ - I^−^, obtained from the pNPD measurements at 5 K. The Rietveld refinement is superimposed on the data and the fit residual is shown below it. **e** The magnetization ellipsoids obtained from the pNPD analysis at 5 K. The Yb^3+^ anisotropy is nearly Ising with a strong tendency for the moments to align close to the crystallographic *c*-axis. **f** Magnetization vs applied magnetic field for polycrystalline Yb_2_Be_2_SiO_7_ at 2 K, 5 K, and 10 K. The measured values at 1.5 T are labeled on the panel.

Zero-field specific heat data measured with a single crystal and the corresponding magnetic entropy extracted by integrating *C*_*p*_/*T* as a function of temperature are shown in Fig. 2a and its inset, respectively. There are no typical features of magnetic ordering in the heat capacity data. Instead, it is dominated by a broad peak centered near 0.5 K, while the magnetic entropy approaches *R*ln(2) at 4 K. The entropy result validates the use of an effective spin-1/2 model for the Yb^3+^ ions at low-*T* and indicates that the broad peak does not have a crystal field origin. Neutron powder spectroscopy, with the scattering intensity plotted as a function of momentum transfer *Q* and energy transfer *E* in Fig. 2b, was used to measure the Yb^3+^ crystal field spectrum. The magnetic scattering contribution was isolated by subtracting the scattering from the non-magnetic analog Lu_2_Be_2_SiO_7_ measured under the same experimental conditions and scaled appropriately to account for the scattering cross-section difference between Yb and Lu. There is no evidence for low-lying crystal field levels in this data. Instead, there are three excitations centered about 11, 23, and 36 meV as shown in Fig. 2c that likely correspond to the three excited crystal field doublets expected for *J* = 7/2 Kramers Yb^3+^ ions in a low-symmetry ligand environment. Interestingly, these three excitations are much broader than the instrument energy resolution, which is readily apparent when comparing this data to previous results from Er_2_Be_2_SiO_7_^51^ with nearly resolution-limited crystal field levels. This broadening may be a consequence of the Be/Si site mixing that we identified in the Rietveld refinements of the HB-2A data. Interestingly, we confirmed that there is no evidence for Be/Si site mixing in the previously reported HB-2A data for Er_2_Be_2_SiO_7_^51^. We also collected Pair-Distribution-Function (PDF) data using the time-of-flight powder diffractometer NOMAD to investigate possible local structural distortions in Yb_2_Be_2_SiO_7_ (see Supplementary Discussion Section I and Supplementary Fig. S1), but the PDF data is explained well by the global structure.

The Yb^3+^ ions have a monoclinic point group symmetry of C_*s*_ with a single mirror plane symmetry element. The crystal field environment of the four Yb^3+^ ions in the chemical unit cell is different, with the *y*-axis of the local coordinate system indicated by the orange arrows in Fig. 1b. The local *y*-axes are parallel to the mirror-plane normals and perpendicular to the intradimer bonds. One principal g-tensor direction lies along the local *y*-axis. The other two principal g-tensor directions are not constrained by symmetry and make an angle of *β* with the crystallographic [001] and [110] or $[1\bar{1}0]$ directions. The local coordinate system sketched in Fig. 1b applies when *β* = 0. Under this assumption, the blue arrows in this same panel denote the local *x*-axes, which are parallel to the dimer bonds.

Due to the low point symmetry of the Yb^3+^ ions and the lack of sufficient crystal field observables in the neutron spectroscopy data, it is not feasible to obtain a unique set of crystal field parameters for Yb_2_Be_2_SiO_7_. Half-polarized neutron powder diffraction (pNPD) offers a powerful alternative to probe the single-ion anisotropy of this system via the local site susceptibility approach^55^. pNPD is performed in the paramagnetic region and therefore the lack of long-range order does not influence the application of this technique. Spin-up and spin-down neutron diffraction patterns with intensities of I^+^ and I^−^ are measured separately, and then Rietveld refinements of the sum and difference patterns (given by I^+^ + I^−^ and I^+^ - I^−^, respectively) are performed using the software CrysPy^55^. The Yb^3+^ local site susceptibility tensor, which provides information on the local anisotropy and principal *g*-tensor directions and their relative magnitudes, can be extracted from the difference pattern.

The difference pattern I^+^ - I^−^ measured at 5 K in an applied magnetic field of 1.5 T is shown in Fig. 2d and the sum pattern from the same dataset is shown in Supplementary Fig. S2. In each case, the Rietveld refinement result is superimposed on the data and the fit residual is plotted below the data. The best-fit result of the difference pattern yields a small *β* value of 1.52(3)^°^, which means that the two principal *g*-tensor directions not constrained by symmetry are nearly aligned with the crystallographic [001]-axis and the dimer bond direction ([110] and $[1\bar{1}0]$ for the two dimer sublattices) as illustrated in Fig. 1b. The magnetization ellipsoids’ principal axes have magnitudes of 2.32 *μ*_*B*_/T, 0.14 *μ*_*B*_/T, and 0.10 *μ*_*B*_/T, with the largest value for the pseudo-[001]-axis direction. These results are indicative of an Ising-like anisotropy with small deviations away from the [001]-axis and they are consistent with our Curie–Weiss fitting results described above. The analysis of the 10 K difference pattern measured in the same 1.5 T magnetic field produces similar results. The magnetization ellipsoids calculated from the local site susceptibility analysis are presented in Fig. 2e. CrysPy can also be used to calculate the powder-averaged magnetization from the local-site susceptibility tensor to facilitate a direct comparison to the experimental data as a sanity check. The powder-averaged magnetization at selected temperatures for Yb_2_Be_2_SiO_7_ is presented in Fig. 2f with the measured 1.5 T values shown on the panel. The 5 K and 10 K values calculated from the refined local site susceptibility tensors are 0.53 *μ*_*B*_/Yb and 0.29 *μ*_*B*_/Yb, respectively, which are nearly the same values from our powder-averaged magnetization measurements.

### Isolated dimer model

**Fig. 3:**
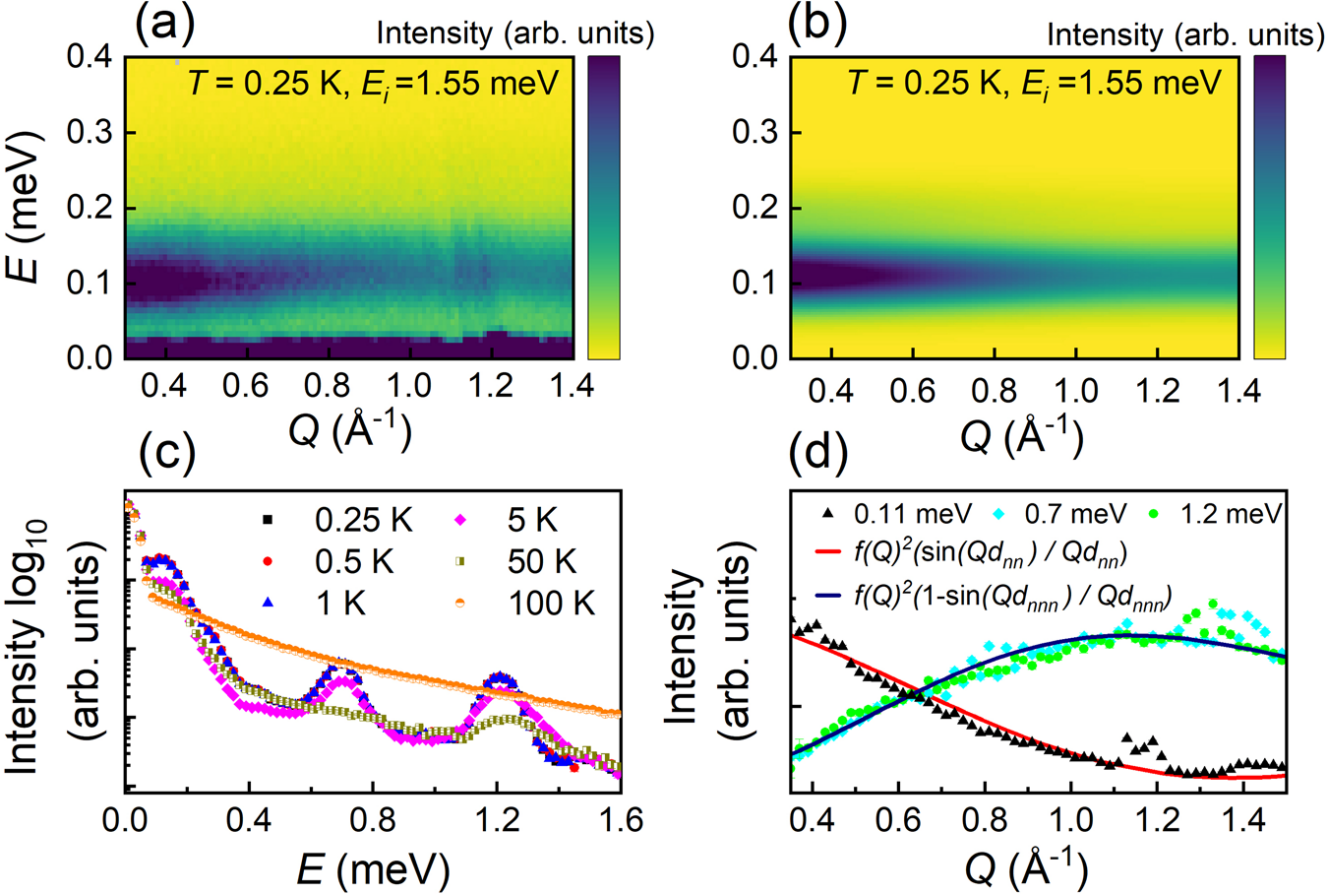
Dynamical structure factor. **a** Color contour plot of the scattering intensity as a function of momentum and energy transfer at 0.25 K from CNCS with *E*_*i*_ = 1.55 meV. A weakly-dispersive mode with a magnetic origin is centered at 0.11 meV. **b** Color contour plot of the simulated scattering intensity using an isolated dimer model with the anisotropic intradimer exchange parameters provided in the main text. **c** Constant-*Q* cuts of the scattering intensity (*Q* integration range [0.5, 1.8] Å^−1^) with *E*_*i*_ = 2.49 meV at selected temperatures. Aside from the main excitation band centered at 0.11 meV with a shoulder at 0.19 meV, there are two higher excitation bands centered at 0.7 meV and 1.2 meV, respectively. **d** Constant-*E* cuts centered about three different mode positions, with an energy integration range ±0.05 meV for the lowest mode (*E*_*i*_ = 1.55 meV data) and an integration range of ±0.1 meV for the two higher modes (*E*_*i*_ = 2.49 meV data). The intensities of the two higher-energy cuts have been rescaled to facilitate a straightforward comparison of their *Q*-dependence, which is strikingly different from the lower excitation band. Simulations based on two different types of single-dimer ground states are superimposed on the data and describe it well.

Low-energy neutron powder spectroscopy data were used to investigate the magnetic excitations associated with the collective ground state of Yb_2_Be_2_SiO_7_. A color contour plot of the scattering intensity as a function of *Q* and *E* with *T* = 0.25 K measured on CNCS with *E*_*i*_ = 1.55 meV is presented in Fig. 3a. A simulated scattering pattern based on an isolated dimer model that will be discussed below with Gaussian peak widths (full-width half maximums) that match the expected instrumental energy resolution at the mode positions (i.e. 0.035 meV at 0.11 meV and 0.033 meV at 0.19 meV) is depicted in Fig. 3b. A low-energy band of excitations with minimal dispersion centered at 0.11 meV and an intensity maximum as *Q* → 0 is visible in the data. There is also a second excitation at 0.19 meV that manifests as a shoulder in the data. Both mode energies were determined by fitting the data to a sum of Gaussian functions as shown in Supplementary Fig. S3 and summarized in Supplementary Table S4. Constant-*Q* cuts at selected temperatures are presented in Supplementary Fig. S4 for the *E*_*i*_ = 1.55 meV dataset and over a wider energy range in Fig. 3c for the *E*_*i*_ = 2.49 meV dataset. The logarithmic intensity scales help to reveal two higher-energy excitation bands centered at 0.7 meV and 1.2 meV. The *Q*-dependence of the three distinct modes is shown in Fig. 3d. Both the *Q* and *T*-dependence of these modes indicate that they are magnetic excitations and their nearly-dispersionless nature suggests that they are associated with isolated or weakly-interacting spin dimers.

Since effective spin-1/2 dimer models with strong intradimer exchange anisotropy can have a maximum of three single-dimer excitations, it is clear that all four modes (at 0.11, 0.19, 0.7 and 1.2 meV) observed here do not have the same origin. We first verified that none of these modes were spurious by measuring them with different incident energies. Zero-field heat capacity only depends on the eigenvalues of these models, so we compared our data shown in Fig. 2a to simulations assuming various energy-level schemes (see Supplementary Fig. S5). The best simulation of the zero-field heat capacity data corresponds to an isolated dimer model with a doubly-degenerate mode at 0.11 meV and a non-degenerate mode at 0.19 meV. This implies that the two higher-energy modes have a different origin. In fact, as shown in Fig. 3d their *Q*-dependence is well-described by the Yb^3+^ magnetic form factor squared multiplied by the known structure factor of a triplet excitation for an isolated Heisenberg dimer model^56^ with the square plaquette distance *d*_*nnn*_ = 3.84 Å. Since these higher-energy modes have a negligible contribution to the specific heat, this implies that only a small fraction of Yb^3+^ ions form longer-range dimers in Yb_2_Be_2_SiO_7_, possibly due to the known Be/Si site mixing in this system. We also compared the *Q*-dependence of these modes to expectations for the lowest-energy excitations of rectangular tetramers^56^, but this model could not account for the *Q*-position of their maximum intensities. Finally, we note that higher-energy excitations in SrCu_2_(BO_3_)_2_ have been attributed to singlet bound states consisting of contributions from multiple dimer units^57–59^ and a similar alternative origin for the higher-energy modes cannot be ruled out in the present case.

With the energy level scheme for a possible isolated dimer model comprised of most Yb^3+^ ions in the system established, we performed a quantitative analysis of the low-energy neutron spectroscopy data and field-dependent heat capacity and magnetization data. We started by constructing a minimal model based on symmetry analysis^46,60^. The model Hamiltonian describes a collection of isolated dimers with anisotropic intradimer interactions and Zeeman coupling:

1 $${{{\mathcal{H}}}}={\sum }_{ < i,j > l\alpha }{S}_{il}^{\alpha } \, {J}_{\alpha \alpha }^{l}{S}_{jl}^{\alpha }-{\mu }_{B}{\sum }_{il\alpha \beta }{H}^{\alpha }{g}_{\alpha \beta }^{l}{S}_{il}^{\beta }$$

where the superscript *l* denotes dimers in sub-lattice A/B, ${S}_{i}^{\alpha }$ refers to the *α*( = *x*, *y*, *z*) component of the effective spin 1/2 operator with *x* = [1, 1, 0], *y* = [ − 1, 1, 0] and *z* = [0, 0, 1], ${J}_{\alpha \alpha }^{l}$ represents one component of the intradimer exchange tensor for dimers in each sub-lattice, *H*^*α*^ is the *α* component of the applied magnetic field, and ${g}_{\alpha \beta }^{l}$ represents one component of the *g*-tensor. Our model is defined in global crystallographic coordinates and it is equivalent to the one defined within the local basis after proper rotations. Constrained by the time-reversal and mirror symmetries, this Hamiltonian takes a diagonal form with an *XYZ* type of spin anisotropy in the reference frame defined above, and the couplings on the two sub-lattices are related by a 90^°^ rotation about the crystallographic [001]-axis so that ${J}_{xx/yy}^{A}={J}_{yy/xx}^{B}$ and ${J}_{zz}^{A}={J}_{zz}^{B}$.

**Fig. 4:**
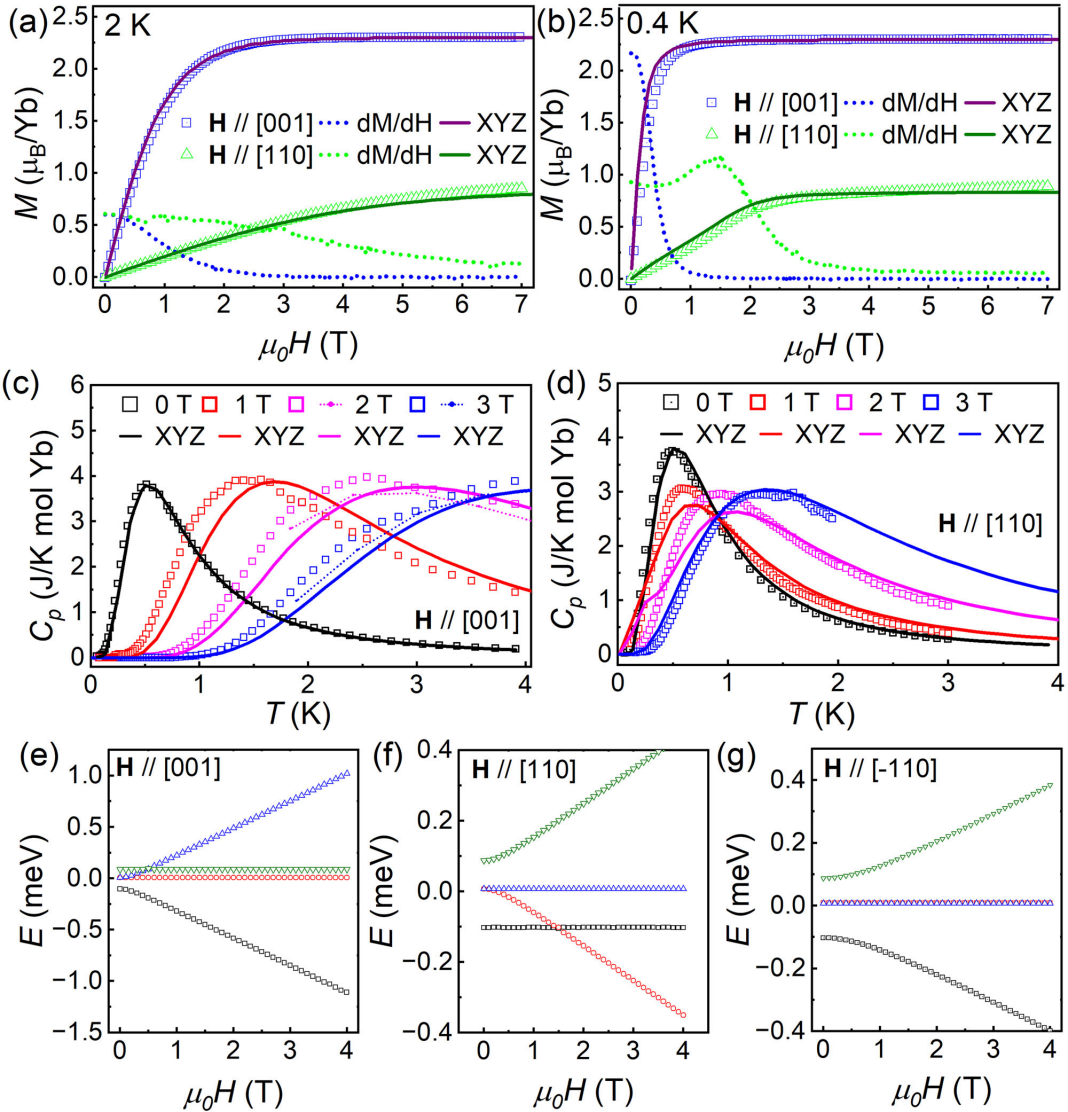
Isolated dimer modeling. **a** Magnetization vs field along two high-symmetry crystallographic directions at 2 K. **b** Similar data collected at 0.4 K. **c** Heat capacity vs magnetic field applied along the [001] direction. The initial experimental data with a modest field misalignment are shown as open symbols, the remeasured 2 T and 3 T data over a limited temperature range are shown as points connected by dashed curves, and the simulated data from our XYZ anisotropic exchange model are shown as solid curves. **d** Heat capacity data vs magnetic field applied along the [110] direction with simulated data from the same model superimposed on it. **e**–**g** Simulated isolated dimer energy levels vs applied field along different crystallographic directions for one dimer sublattice based on the XYZ anisotropic exchange model. There is a level crossing near 1.5 T for one dimer sublattice when **H**∥[110] or for the other dimer sublattice when ${{{\bf{H}}}}\parallel [\bar{1}10]$.

Although off-diagonal *g*-tensor components are allowed by symmetry, our pNPD results described above suggest they are much smaller than the diagonal ones. We hence take the approximation that the g-tensor follows the same symmetry as the exchange tensor. With the principal *g*-tensor directions now fixed along high-symmetry crystallographic directions, we estimated the *g*-tensor values by using the 2 K magnetization data, with the **H**∥[001] and **H**∥[110] measurements presented in Fig. 4a. After subtracting off a linear van-Vleck contribution in the highest-field regime, we obtain ${g}_{xx}^{A}=1.64$, ${g}_{yy}^{A}=1.71$, and ${g}_{zz}^{A}=4.6$.

The energy level scheme established from the neutron spectroscopy and zero-field heat capacity data described above is consistent with 12 different intradimer exchange matrices, which we determined using Supplementary Eq. (S2). We then performed exact diagonalization of these 12 Hamiltonians in zero field to find six solutions with *S*_*z*_ = 0 ground state wavefunctions and six solutions with *S*_*z*_ ≠ 0 ground state wavefunctions. We calculated the dimer structure factors for the three excitations of each model using Supplementary Eq. (S4), with the results for three representative models shown in Supplementary Fig. S6. Importantly, all 12 models have one intense mode and two much weaker modes. This common feature allows us to reliably compare the *Q*-dependence of the intense mode for the models with the neutron spectroscopy data even though neither weak mode is completely resolved from the intense one. The two types of solutions are characterized by intense dimer excitations with very different *Q*-dependences. In the regime *Q* ≤ 1.6 Å^−1^, the intense excitation for the *S*_*z*_ = 0 solutions is described by the structure factor,

2 $$S(Q)=A\left(1-\frac{sin(Qd)}{Qd}\right)$$

where *A* is a constant and *d* is the intradimer distance. In the same *Q*-regime, the intense excitation for the *S*_*z*_ ≠ 0 solutions is described approximately by the structure factor

3 $$S(Q)\approx A\frac{sin(Qd)}{Qd}.$$

To narrow down possible intradimer exchange matrices, we examined the *Q*-dependence of the constant *E*-cut centered at the intense 0.11 meV mode with an integration range of ±0.05 meV shown in Fig. 3d. The data fits well to the expected structure factor for the intense mode of the six *S*_*z*_ ≠ 0 solutions. Two of these options can be ruled out because their most intense mode is located at 0.19 meV, which is inconsistent with the neutron spectroscopy data. This leaves four viable solutions and the corresponding simulations for them are presented in Supplementary Fig. S7.

All four remaining candidate models have single-dimer ground states of the form $(\left|\uparrow \uparrow \right\rangle+\left|\downarrow \downarrow \right\rangle )/\sqrt{2}$ or $(\left|\uparrow \uparrow \right\rangle -\left|\downarrow \downarrow \right\rangle )/\sqrt{2}$ with a dominant contribution from the other *S*_*z*_ ≠ 0 wavefunction to the doubly-degenerate first excited state. We note that these two dimer ground states are qualitatively consistent with the low-*T* magnetic susceptibility data presented in Fig. 1d, which should level off at finite values for all field orientations as *T* → 0 rather than drop to zero as expected for a Heisenberg dimer model. Two of the solutions show superior agreement with the bulk characterization data. Although the *Q*-dependence of the weak mode intensities from neutron powder spectroscopy is not exactly the same for these two models, our data is not sufficient for differentiating between them conclusively. The exchange parameters for one of these models are *J*_*xx*_ = 0.19 meV, *J*_*yy*_ = − 0.03 meV, and *J*_*zz*_ = − 0.19 meV and the exchange parameters for the second model are *J*_*xx*_ = − 0.03 meV, *J*_*yy*_ = 0.19 meV, and *J*_*zz*_ = − 0.19 meV.

Using the Hamiltonian parameters provided above for the first model, the neutron spectroscopy simulation is shown in Fig. 3b and the calculated single crystal magnetization and heat capacity data are superimposed on the corresponding experimental datasets in Fig. 4a–d. It is worth noting that there is no evidence for field-induced magnetic order in the heat capacity measurements down to dilution fridge temperatures. The agreement between experiment and theory is excellent, with the small differences between the **H**∥[001] heat capacity data and the simulation arising from modest sample misalignment that was confirmed by re-measuring the 2 T and 3 T data with coarse temperature steps for *T* ≥ 1.8 K, which are shown as points connected by dashed curves in Fig. 4c. This simulation yields an effective *S* = 1 dimer state $(\left|\uparrow \uparrow \right\rangle -\left|\downarrow \downarrow \right\rangle )/\sqrt{2}$ in zero field, which is stabilized by the large ferromagnetic exchange interaction *J*_*zz*_ along the quantization axis. The excited states are doubly degenerate $(\left|\uparrow \uparrow \right\rangle+\left|\downarrow \downarrow \right\rangle )/\sqrt{2}$ and $(\left|\uparrow \downarrow \right\rangle -\left|\downarrow \uparrow \right\rangle )/\sqrt{2}$ at *E* = 0.11 meV and $(\left|\uparrow \downarrow \right\rangle+\left|\downarrow \uparrow \right\rangle )/\sqrt{2}$ at *E* = 0.19 meV, respectively. The second model has a similar eigenvector scheme for its modes, although the two *S*_*z*_ ≠ 0 modes swap their eigenvalues so the ground state is $(\left|\uparrow \uparrow \right\rangle+\left|\downarrow \downarrow \right\rangle )/\sqrt{2}$ instead. Both isolated dimer models account for the major experimental features well, including the *Q* and *E*-dependence of the dynamical structure factor for the dimer excitations and the temperature and magnetic-field-dependence of the magnetization and specific heat. These results confirm that the interdimer interactions are significantly weaker than the intradimer interactions in Yb_2_Be_2_SiO_7_.

Beyond the qualitative signatures of the *S*_*z*_ ≠ 0 dimer ground state in neutron spectroscopy, we also observe an anisotropic magnetization process that is not expected for systems with *S*_*z*_ = 0 dimer ground states. In particular, no low-field magnetization plateaus are observed at 0.4 K when the magnetic field is applied along any high-symmetry crystallographic direction, which is in sharp contrast to expectations for *S*_*z*_ = 0 dimer ground states as shown in Supplementary Fig. S6. Instead, applying a magnetic field along the [001] direction continuously rotates the $(\left|\uparrow \uparrow \right\rangle -\left|\downarrow \downarrow \right\rangle )/\sqrt{2}$ ground state without closing the excitation gap so that the system is immediately magnetized and evolves to the fully-polarized state adiabatically without a transition as shown in the Fig. 4b data and illustrated by the energy level vs field diagram presented in Fig. 4e. On the other hand, applying the field along the [110] direction causes a level crossing for one dimer sublattice as shown in Fig. 4f, which corresponds to a quantum phase transition to an intermediate state as indicated by the peak in *dM*/*dH* shown in Fig. 4b. The other dimer sublattice has a similar level crossing when ${{{\bf{H}}}}\parallel [\bar{1}10]$. It is worth noting that the largest discrepancy in the **H**∥ [110] bulk characterization data and the simulations is observed in the fixed-field heat capacity measurements shown in Fig. 4d near the expected critical field for the level crossing. This is likely due to neglecting the appropriate sub-leading interaction in the simulations, which should have the largest effect on the magnetic properties of the system in this regime^46^. Indeed, neutron powder spectroscopy also suggests that a small additional term should be added to the Hamiltonian to capture the increased bandwidth of the 0.11 meV mode observed in the data, but its accurate determination without single-crystal neutron spectroscopy data is challenging. The nature of this term is not obvious either, as the expected sub-1 K energy scale means it could arise from interdimer exchange or a dipolar interaction. While additional theoretical work on candidate magnetic Hamiltonians for this system and its isostructural family members could help to address this issue, neglecting this extra term does not affect the exotic zero-field magnetic ground state for Yb_2_Be_2_SiO_7_ established here.

## Discussion

Quantum dimer magnets with pure spin-1/2 degrees of freedom have been studied extensively^4,8^. Antiferromagnetic Heisenberg exchange at the intradimer level stabilizes the entangled singlet ground state $(\left|\uparrow \downarrow \right\rangle -\left|\downarrow \uparrow \right\rangle )/\sqrt{2}$ in zero field with a spin gap to a triplet excited state. Key experimental signatures of this exotic ground state are an isotropic decrease in the magnetic susceptibility with decreasing temperature and zero magnetization (i.e., a magnetization plateau) at sufficiently low temperatures and applied fields, along with a powder-averaged dynamical structure factor for the triplet excitation that satisfies Eq. (2) exactly^56^. Quantum dimer magnets with effective spin-1/2 degrees of freedom can exhibit very different behavior due to their realization of anisotropic exchange Hamiltonians. The type of magnetic ion and its point symmetry, along with the composition of the crystal-field ground-state wavefunctions, can give rise to intradimer exchange matrices with different symmetries including Ising, XY, XXZ, or even XYZ. The quasi-orthorhombic point symmetry for the Yb^3+^ ions in Yb_2_Be_2_SiO_7_ generates the XYZ dimer model, which was only recently explored by theorists^60^. Their most important insight was that the ground state of the XYZ model can be any one of the four possible dimer states depending on the sign and relative strength of the three anisotropic exchange matrix terms. One can distinguish between the four dimer ground states via qualitatively different magnetic susceptibilities, magnetizations, and powder-averaged dynamical structure factors, as shown in Supplementary Fig. S6. We have measured all three quantities for Yb_2_Be_2_SiO_7_ and found that their behavior matches expectations for the novel $(\left|\uparrow \uparrow \right\rangle -\left|\downarrow \downarrow \right\rangle )/\sqrt{2}$ or $(\left|\uparrow \uparrow \right\rangle+\left|\downarrow \downarrow \right\rangle )/\sqrt{2}$ ground state.

Our results have broad implications for other quantum dimer magnets with effective spin-1/2 degrees of freedom and show that anisotropic intradimer exchange should always be considered when trying to understand the magnetic properties of these materials. Yb_2_Si_2_O_7_ was the first insulating rare-earth-based quantum dimer magnet discovered with effective spin-1/2 moments^61^. While the bulk characterization and neutron scattering results are broadly consistent with a $(\left|\uparrow \downarrow \right\rangle -\left|\downarrow \uparrow \right\rangle )/\sqrt{2}$ zero-field ground state, the magnetic Hamiltonian is still unknown. An interacting version of the XYZ dimer model discussed here may be appropriate due to the low-point symmetry of the Yb ions in that system. Anisotropic intradimer exchange should also be important for the new insulating SSL families Ba*R*_2_M*X*_5_ (*R* = rare earth; *M* = Zn, Pd, or Pt; *X* = O or S) and *R*_2_Be_2_*Z*O_7_ (*Z* = Ge or Si). In fact, it has been shown to stabilize the same $(\left|\uparrow \uparrow \right\rangle -\left|\downarrow \downarrow \right\rangle )/\sqrt{2}$ dimer ground state in the effective spin-1/2 system BaCe_2_ZnS_5_^46^ that we have identified as one possibility for Yb_2_Be_2_SiO_7_ here. Despite this progress, direct links between models with anisotropic intradimer exchange and the measured magnetic properties of these materials have remained scarce. There is now ample materials’ phase space to explore this topic in more detail.

In summary, we have characterized the low-temperature magnetic properties of the quantum dimer magnet Yb_2_Be_2_SiO_7_ using bulk characterization and neutron scattering techniques. We find that the effective spin-1/2 Yb^3+^ moments form an unconventional, bipartite entangled state arising from dominant, anisotropic intradimer exchange. The magnetic Hamiltonian for Yb_2_Be_2_SiO_7_ gives rise to a dimer ground state with a *S*_*z*_ ≠ 0 ground state wavefunction instead of the $(\left|\uparrow \downarrow \right\rangle -\left|\downarrow \uparrow \right\rangle )/\sqrt{2}$ state commonly realized in Heisenberg dimer systems based on spin-1/2 moments. A significant amount of Be/Si site mixing in Yb_2_Be_2_SiO_7_ may give rise to the broad crystal field levels and possibly the two higher-energy dimer excitations. Our work shows that quantum dimer magnets with strong spin-orbit coupling are promising playgrounds for identifying unusual entangled states of matter in quantum materials.

## Methods

### Sample preparation

Polycrystalline samples of Yb_2_Be_2_SiO_7_ were prepared from stoichiometric amounts of Yb_2_O_3_, BeO, and SiO_2_ as detailed in ref. ^48^. Small single crystals were grown using the floating zone melt method^62,63^ and oriented via Laue back-diffraction^64,65^. Detailed structure refinements of single-crystal x-ray diffraction data (XRD) agree well with the previously-reported structure obtained from powder XRD^48^ and the results are provided in Supplementary Tables S1, S2.

### Bulk characterization

Polycrystalline and single-crystal DC magnetization measurements as a function of magnetic field and temperature were performed using an MPMS3 SQUID magnetometer (Quantum Design) equipped with a He-3 insert. For the single crystal measurements, the magnetic fields were applied along high-symmetry crystallographic directions. Specific heat measurements using single crystals were performed with a Quantum Design Physical Property Measurement System (PPMS) equipped with a dilution refrigerator insert. The AC susceptibility measurements were conducted on single crystals with a voltage-controlled current source (Stanford Research, CS580) and lock-in amplifier (Stanford Research, SR830). The phase of the lock-in amplifier was set to measure the first harmonic signal. The RMS amplitude of the AC excitation field was set to be 0.6 Oe with the frequency fixed to be 200 Hz. The measurements were performed in the SCM1 Dilution Refrigerator of the National High Magnetic Field Laboratory, Tallahassee.

### Neutron scattering

Neutron powder diffraction (NPD) was performed using the high-resolution HB-2A powder diffractometer^66,67^ at the High Flux Isotope Reactor (HFIR) of Oak Ridge National Laboratory (ORNL). Unpolarized measurements were conducted with a ~4 g polycrystalline sample sealed inside an aluminum can with 1 atm of helium exchange gas. Diffraction patterns were collected at 250 mK and 2 K with a wavelength of 2.41 Å and a collimation of open-open-12’.

A half-polarized neutron powder diffraction (pNPD) experiment was performed using the same HB-2A collimation settings and a V-cavity to generate the polarized beam with a ~13 g polycrystalline sample pressed into pellets. The sample was loaded in a larger Al can than the one used for the unpolarized measurements. The polarization state was controlled using a Mezei flipper. Further details of the HB-2A pNPD experimental set-up can be found in ref. ^68^. Spin-up and spin-down diffraction patterns, with intensities denoted by I^+^ and I^−^ respectively, were collected in a vertical magnetic field of 1.5 T at temperatures of 5 and 10 K using a wavelength of 2.41 Å.

Time-of-flight neutron powder diffraction data were collected at 100 K on NOMAD^67^ at the Spallation Neutron Source (SNS) of ORNL using ~4 g of powder. Similar measurements were carried out on ~4.5 g of crushed single crystals and ~2.3 g of polycrystalline Er_2_Be_2_SiO_7_^51^.

Neutron powder spectroscopy data were obtained from the direct-geometry time-of-flight instrument SEQUOIA^69^ at the SNS using ~2.5 g of similar Yb_2_Be_2_SiO_7_ powder. The same amount of a non-magnetic reference sample Lu_2_Be_2_SiO_7_ was also measured. All SEQUOIA data were collected at 5 K with an incident energy of 80 meV using the fine Fermi chopper. The *T*_0_ frequency, Fermi chopper frequency, and energy resolution at the elastic line (full-width half-maximum) were 90 Hz, 480 Hz, and 2.15 meV.

Lower-incident-energy neutron powder spectroscopy data were measured with the direct-geometry time-of-flight instrument CNCS^70^ at the SNS using the same polycrystalline sample from the unpolarized HB-2A experiment. The CNCS data were collected using incident energies of *E*_*i*_ = 1.55 meV and 2.49 meV in the “high flux” chopper setting mode, which produced energy resolutions of 0.04 meV and 0.06 meV (full-width half-maximum) at the elastic line, respectively.

## Supplementary information

Supplementary Information

Peer Review File

## Source data

Source Data

## Data Availability

All the data supporting the findings of this study are available within the article and from the corresponding authors upon request. Source data are provided with this paper.

## References

[1] Sachdev, S. Quantum magnetism and criticality. *Nat. Phys.* **4**, 173–185 (2008).

[2] Balents, L. Spin liquids in frustrated magnets. *Nature* **464**, 199–208 (2010). 20220838 10.1038/nature08917

[3] Bose, I. Spin gap antiferromagnets: materials and phenomena. *Curr. Sci.* **88**, 62–70 (2005).

[4] Zapf, V., Jaime, M. & Batista, C. D. Bose-Einstein condensation in quantum magnets. *Rev. Mod. Phys.* **86**, 563–614 (2014).

[5] Sriram Shastry, B. & Sutherland, B. Exact ground state of a quantum mechanical antiferromagnet. *Phys. B+C.* **108**, 1069–1070 (1981).

[6] Liu, H.-D., Chen, Y.-H., Lin, H.-F., Tao, H.-S. & Liu, W.-M. Antiferromagnetic metal and Mott transition on Shastry-Sutherland lattice. *Sci. Rep.* **4**, 4829 (2014). 24777282 10.1038/srep04829 PMC4003478

[7] Romhányi, J., Penc, K. & Ganesh, R. Hall effect of triplons in a dimerized quantum magnet. *Nat. Commun.* **6**, 6805 (2015). 25865559 10.1038/ncomms7805

[8] Giamarchi, T., Rüegg, C. & Tchernyshyov, O. Bose-Einstein condensation in magnetic insulators. *Nat. Phys.* **4**, 198–204 (2008).

[9] Kageyama, H., Onizuka, K., Yamauchi, T. & Ueda, Y. Crystal growth of the two-dimensional spin gap system SrCu_2_(BO_3_)_2_. *J. Cryst. Growth* **206**, 65–67 (1999).

[10] Kageyama, H. et al. Exact dimer ground state and quantized magnetization plateaus in the two-dimensional spin system SrCu_2_(BO_3_)_2_. *Phys. Rev. Lett.* **82**, 3168–3171 (1999).

[11] Miyahara, S. & Ueda, K. Exact dimer ground state of the two dimensional Heisenberg spin system SrCu_2_(BO_3_)_2_. *Phys. Rev. Lett.* **82**, 3701–3704 (1999).

[12] Weihong, Z., Hamer, C. J. & Oitmaa, J. Series expansions for a Heisenberg antiferromagnetic model for SrCu_2_(BO_3_)_2_. *Phys. Rev. B* **60**, 6608–6616 (1999).

[13] Zayed, M. E. et al. 4-spin plaquette singlet state in the Shastry-Sutherland compound SrCu_2_(BO_3_)_2_. *Nat. Phys.* **13**, 962–966 (2017).

[14] Wang, L., Zhang, Y. & Sandvik, A. W. Quantum spin liquid phase in the Shastry-Sutherland model detected by an improved level spectroscopic method. *Chin. Phys. Lett.* **39**, 077502 (2022).

[15] Yang, J., Sandvik, A. W. & Wang, L. Quantum criticality and spin liquid phase in the Shastry-Sutherland model. *Phys. Rev. B* **105**, L060409 (2022).

[16] Corboz, P., Zhang, Y., Ponsioen, B. & Mila, F. Quantum spin liquid phase in the Shastry-Sutherland model revealed by high-precision infinite projected entangled-pair states. Preprint at https://arxiv.org/abs/2502.14091 (2025). 10.1103/67xm-5d8v 42172416

[17] Viteritti, L. L., Rende, R., Parola, A., Goldt, S. & Becca, F. Transformer wave function for two dimensional frustrated magnets: emergence of a spin-liquid phase in the Shastry-Sutherland model. *Phys. Rev. B* **111**, 134411 (2025).

[18] Kodama, K. et al. Magnetic superstructure in the two-dimensional quantum antiferromagnet SrCu_2_(BO_3_)_2_. *Science* **298**, 395–399 (2002). 12376697 10.1126/science.1075045

[19] Sebastian, S. E. et al. Fractalization drives crystalline states in a frustrated spin system. *Proc. Natl. Acad. Sci.* **105**, 20157–20160 (2008). 19074281 10.1073/pnas.0804320105 PMC2629284

[20] Jaime, M. et al. Magnetostriction and magnetic texture to 100.75 Tesla in frustrated SrCu_2_(BO_3_)_2_. *Proc. Natl. Acad. Sci.* **109**, 12404–12407 (2012).

[21] Corboz, P. & Mila, F. Crystals of bound states in the magnetization plateaus of the Shastry-Sutherland model. *Phys. Rev. Lett.* **112**, 147203 (2014). 24766008 10.1103/PhysRevLett.112.147203

[22] Meng, Z. Y. & Wessel, S. Phases and magnetization process of an anisotropic Shastry-Sutherland model. *Phys. Rev. B* **78**, 224416 (2008).

[23] Suzuki, T., Tomita, Y., Kawashima, N. & Sengupta, P. Finite-temperature phase transition to the *m* = 1/2 plateau phase in the spin-1/2 XXZ model on the Shastry-Sutherland lattices. *Phys. Rev. B* **82**, 214404 (2010).

[24] Dublenych, Y. I. Ground states of the Ising model on the Shastry-Sutherland lattice and the origin of the fractional magnetization plateaus in rare-earth-metal tetraborides. *Phys. Rev. Lett.* **109**, 167202 (2012). 23215119 10.1103/PhysRevLett.109.167202

[25] Su, L., Wierschem, K. & Sengupta, P. Magnetic phases in the *S* = 1 Shastry-Sutherland model with uniaxial anisotropy. *Phys. Rev. B* **89**, 245432 (2014).

[26] Zhang, Z. & Sengupta, P. Generalized plaquette state in the anisotropic Shastry-Sutherland model. *Phys. Rev. B* **92**, 094440 (2015).

[27] Yadav, L. et al. Observation of unprecedented fractional magnetization plateaus in a new Shastry-Sutherland Ising compound. *Phys. Rev. X* **15**, 041045 (2025).

[28] Michimura, S. et al. Magnetic frustrations in the Shastry-Sutherland system ErB_4_. *Phys. B Condens. Matter* **378-380**, 596–597 (2006).

[29] Brunt, D. et al. Magnetisation process in the rare earth tetraborides, NdB_4_ and HoB_4_. *Sci. Rep.* **8**, 232 (2018). 29321474 10.1038/s41598-017-18301-1 PMC5762811

[30] Yoshii, S. et al. Multistep magnetization plateaus in the Shastry-Sutherland system TbB_4_. *Phys. Rev. Lett.* **101**, 087202 (2008). 18764654 10.1103/PhysRevLett.101.087202

[31] Wierschem, K. et al. Origin of modulated phases and magnetic hysteresis in TmB_4_. *Phys. Rev. B* **92**, 214433 (2015).

[32] Orendác, M. et al. Ground state and stability of the fractional plateau phase in metallic Shastry-Sutherland system TmB_4_. *Sci. Rep.* **11**, 6835 (2021). 33767331 10.1038/s41598-021-86353-5 PMC7994547

[33] Mat’as, S. et al. Magnetism of rare earth tetraborides. *J. Phys. Conf. Ser.* **200**, 032041 (2010).

[34] Feng, J. J. et al. The main 1/2 magnetization plateau in Shastry-Sutherland magnets: effect of the long-range Ruderman-Kittel-Kasuya-Yosida interaction. *Europhys. Lett.* **105**, 17009 (2014).

[35] Wierschem, K., Zhang, Z., Wibawa, A. & Sengupta, P. Magnetization plateaus and supersolid phases in an extended Shastry-Sutherland model. *Eur. Phys. J. B* **91**, 201 (2018).

[36] Farkasovský, P. & Regeciová, L. Magnetization plateaus and phase diagrams of the extended Ising model on the Shastry-Sutherland lattice: Effects of long-range interactions. *Eur. Phys. J. B* **92**, 33 (2019).

[37] Regeciová, L. & Farkasovský, P. Magnetic phase diagram of the Ising model with the long-range RKKY interaction. *Eur. Phys. J. B* **92**, 184 (2019).

[38] Regeciová, L. & Farkasovský, P. Formation of magnetization plateaus in the 3D Ising model with the long-range RKKY interaction: application to rare-earth tetraborides. *Eur. Phys. J. B* **93**, 110 (2020).

[39] Wakeshima, M. et al. Specific heat and neutron diffraction study on quaternary sulfides BaNd_2_CoS_5_ and BaNd_2_ZnS_5_. *J. Solid State Chem.* **174**, 159–164 (2003).

[40] Ozawa, T. et al. Spin frustration and antiferromagnetic long range order in *R*_2_BaPdO_5_ (*R* = rare earth). *Phys. Lett. A* **337**, 130–134 (2005).

[41] Ozawa, T. et al. Magnetization and specific heat measurement of the Shastry-Sutherland lattice compounds: *L**n*_2_BaPdO_5_ (*L**n* = La, Pr, Nd, Sm, Eu, Gd, Dy, Ho). *J. Alloy. Compd.* **448**, 96–103 (2008).

[42] Ishii, Y. et al. High-pressure synthesis, crystal structure, and magnetic properties of the Shastry-Sutherland lattice oxides Ba*L**n*_2_ZnO_5_ (*L**n* = Pr, Sm, Eu). *J. Solid State Chem.* **289**, 121489 (2020).

[43] Billingsley, B. R., Marshall, M., Shu, Z., Cao, H. & Kong, T. Single crystal synthesis and magnetic properties of a Shastry-Sutherland lattice compound BaNd_2_ZnS_5_. *Phys. Rev. Mater.* **6**, 104403 (2022).

[44] Marshall, M. et al. Field-induced partial disorder in a Shastry-Sutherland lattice. *Nat. Commun.* **14**, 3641 (2023). 37336899 10.1038/s41467-023-39409-1 PMC10279730

[45] Pasco, C. M. et al. Anisotropic magnetism of the Shastry-Sutherland lattice material BaNd_2_PtO_5_. *Phys. Rev. Mater.* **7**, 074407 (2023).

[46] Ma, Q. et al. Quantum entanglement of XY-type spin dimers in Shastry-Sutherland lattice. Preprint at https://arxiv.org/abs/2412.17913 (2024). 10.1103/gl65-17z7 42705297

[47] Ashtar, M. et al. Structure and magnetic properties of melilite-type compounds *R**E*_2_Be_2_GeO_7_ (*R**E* = Pr, Nd, Gd-Yb) with rare-earth ions on Shastry-Sutherland lattice. *Inorg. Chem.* **60**, 3626–3634 (2021). 33635649 10.1021/acs.inorgchem.0c03131

[48] Brassington, A., Huang, Q., Aczel, A. A. & Zhou, H. D. Synthesis and magnetic properties of the Shastry-Sutherland family *R*_2_Be_2_SiO_7_ (*R* = Nd, Sm, Gd-Yb). *Phys. Rev. Mater.* **8**, 014005 (2024).

[49] Ishii, Y. et al. Magnetic properties of the Shastry-Sutherland lattice material BaNd_2_ZnO_5_. *Phys. Rev. Mater.* **5**, 064418 (2021).

[50] Song, F. et al. Tunable magnetism and large inverse magnetocaloric effect in Shastry-Sutherland compounds Tb_2_Be_2_Si_1−*x*_Ge_*x*_O_7_ (0 ≤ x ≤ 1). *J. Alloy. Compd.* **960**, 170657 (2023).

[51] Brassington, A. et al. Magnetic properties of the quasi-XY Shastry-Sutherland magnet Er_2_Be_2_SiO_7_. *Phys. Rev. Mater.* **8**, 094001 (2024).

[52] Pula, M. et al. Candidate for a quantum spin liquid ground state in the Shastry-Sutherland lattice material Yb_2_Be_2_GeO_7_. *Phys. Rev. B* **110**, 014412 (2024).

[53] Liu, A. et al. Large magnetocaloric effect in the Shastry-Sutherland lattice compound Yb_2_Be_2_GeO_7_ with spin-disordered ground state. *Phys. Rev. B* **110**, 144445 (2024).

[54] Rodríguez-Carvajal, J. Recent advances in magnetic structure determination by neutron powder diffraction. *Phys. B Condens. Matter* **192**, 55–69 (1993).

[55] Kibalin, I. A. & Gukasov, A. Local magnetic anisotropy by polarized neutron powder diffraction: application of magnetically induced preferred crystallite orientation. *Phys. Rev. Res.* **1**, 033100 (2019).

[56] Haraldsen, J. T., Barnes, T. & Musfeldt, J. L. Neutron scattering and magnetic observables for *S* = 1/2 spin clusters and molecular magnets. *Phys. Rev. B* **71**, 064403 (2005).

[57] McClarty, P. A. et al. Topological triplon modes and bound states in a Shastry-Sutherland magnet. *Nat. Phys.* **13**, 736–741 (2017).

[58] Wulferding, D. et al. Thermally populated versus field-induced triplon bound states in the Shastry-Sutherland lattice SrCu_2_(BO_3_)_2_. *npj Quantum Mater.* **6**, 102 (2021).

[59] Fogh, E. et al. Field-induced bound-state condensation and spin-nematic phase in SrCu_2_(BO_3_)_2_ revealed by neutron scattering up to 25.9 T. *Nat. Commun.* **15**, 442 (2024). 38200029 10.1038/s41467-023-44115-z PMC10781965

[60] Liu, C., Duan, G. & Yu, R. Theory of rare-earth Kramers magnets on a Shastry-Sutherland lattice: Dimer phases in presence of strong spin-orbit coupling. *npj Quantum Mater.* **10**, 109 (2025).

[61] Hester, G. et al. Novel strongly spin-orbit coupled quantum dimer magnet: Yb_2_Si_2_O_7_. *Phys. Rev. Lett.* **123**, 027201 (2019). 31386489 10.1103/PhysRevLett.123.027201

[62] Rey-García, F., Ibáñez, R., Angurel, L. A., Costa, F. M. & de la Fuente, G. F. Laser floating zone growth: overview, singular materials, broad applications, and future perspectives. *Crystals* **11**, 38 (2021).

[63] Koohpayeh, S., Fort, D. & Abell, J. The optical floating zone technique: a review of experimental procedures with special reference to oxides. *Prog. Cryst. Growth Charact. Mater.* **54**, 121–137 (2008).

[64] Greilinger, A. B. A back-reflection Laue method for determining crystal orientation. *Z. fur Kristallographie Crystalline Mater.* **91**, 424–432 (1935).

[65] Warren, B. E. X-ray diffraction methods. *J. Appl. Phys.* **12**, 375–384 (1941).

[66] Garlea, V. O. et al. The high-resolution powder diffractometer at the High Flux Isotope Reactor. *Appl. Phys. A* **99**, 531–535 (2010).

[67] Calder, S. et al. A suite-level review of the neutron powder diffraction instruments at Oak Ridge National Laboratory. *Rev. Sci. Instrum.* **89**, 092701 (2018). 30278771 10.1063/1.5033906

[68] Baral, R. et al. Magnetic pair distribution function and half polarized neutron powder diffraction at the HB-2A powder diffractometer. *J. Magn. Magn. Mater.* **630**, 173342 (2025).

[69] Granroth, G. E. et al. SEQUOIA: A newly operating chopper spectrometer at the SNS. *J. Phys. Conf. Ser.* **251**, 012058 (2010).

[70] Ehlers, G., Podlesnyak, A. A. & Kolesnikov, A. I. The cold neutron chopper spectrometer at the Spallation Neutron Source - a review of the first 8 years of operation. *Rev. Sci. Instrum.* **87**, 093902 (2016). 27782573 10.1063/1.4962024

