# Supplementary material for: Unconventional bipartite entanglement in the quantum dimer magnet Yb2Be2SiO7
Source: Nat Commun. 2026 Mar 25;17:2751. doi: 10.1038/s41467-026-69258-7 (PMC13018291; doi:10.1038/s41467-026-69258-7)
Supplement: Supplementary file 1 — Supplementary Information [file 41467_2026_69258_MOESM1_ESM.pdf]

Supplemental Information: Unconventional bipartite entanglement in the quantum dimer magnet $Yb_2Be_2SiO_7$

A. Brassington,[1] Q. Ma,[2] G. Duan,[3] S. Calder,[2] A.I. Kolesnikov,[2] K.M. Taddei,[2] G. Sala,[4] E.S. Choi,[5] H. Wang,[6] W. Xie,[6] B.A. Frandsen,[7] N. Li,[8] X.F. Sun,[8] C. Liu,[9] R. Yu,[3,10] H.D. Zhou,[1,*] and A.A. Aczel[2,†]

[1]*Department of Physics and Astronomy, University of Tennessee, Knoxville, TN 37996, USA*
[2]*Neutron Scattering Division, Oak Ridge National Laboratory, Oak Ridge, TN 37831, USA*
[3]*School of Physics and Beijing Key Laboratory of Optoelectronic Functional Materials and Micro-nano Devices, Renmin University of China, Beijing 100872, China*
[4]*Oak Ridge National Laboratory, Oak Ridge, TN 37831, USA*
[5]*National High Magnetic Field Laboratory and Department of Physics, Florida State University, Tallahassee, Florida 32310, USA*
[6]*Department of Chemistry, Michigan State University, East Lansing, Michigan 48824, United States*
[7]*Department of Physics and Astronomy, Brigham Young University, Provo, UT 84602, USA*
[8]*Anhui Provincial Key Laboratory of Magnetic Functional Materials and Devices, Institutes of Physical Science and Information Technology, Anhui University, Hefei, Anhui 230601, People's Republic of China*
[9]*School of Engineering, Dali University, Dali, Yunnan 671003, China*
[10]*Key Laboratory of Quantum State Construction and Manipulation (Ministry of Education), Renmin University of China, Beijing 100872, China*

I. STRUCTURAL CHARACTERIZATION

The phase purity of the $Yb_2Be_2SiO_7$ polycrystalline and single crystal samples was confirmed via room-temperature powder x-ray diffraction (XRD) using a HUBER imaging plate Guinier camera 670 with Cu radiation (λ = 1.54059 Å). The single crystal samples were first ground into a fine powder for this measurement. The XRD refinements were performed with the software package FULLPROF [1] using the structure of $Y_2Be_2SiO_7$ [2] as a starting reference.

As an additional check, single crystal XRD measurements were carried out on a Bruker Eco Quest X-ray Diffractometer using Mo radiation (λ = 0.71073 Å) at 300 K and the structure refinement was completed using the Bruker SHELXTL Software Package. The main results are presented in Supplementary Tables S1 and S2 and agree well with the previous powder refinement [3].

Neutron powder diffraction was performed using the high-resolution HB-2A powder diffractometer at the High Flux Isotope Reactor of Oak Ridge National Laboratory. The experimental details are provided in the main article. The 0.25 K and 2 K diffraction patterns are well-described by the known room-temperature crystal structure. The refined lattice parameters at 0.25 K are $a = b = 7.2155(2)$ Å and $c = 4.7171(2)$ Å, which implies that there is some sample-dependence particularly in the basal-plane values. The fractional coordinates at 0.25 K are presented in Supplementary Table S3.

Time-of-flight neutron powder diffraction data were collected at 100 K using the NOMAD beam line at the Spallation Neutron Source on three samples: polycrystalline $Yb_2Be_2SiO_7$, finely ground single crystals of $Yb_2Be_2SiO_7$, and polycrystalline $Er_2Be_2SiO_7$. The goal of this experiment was to identify evidence for local structural distortions that could be responsible for the Yb^{3+} crystal field broadening observed in the SEQUOIA data presented in the main text. The NOMAD data reduction was completed with the Advanced Diffraction Environment (ADDIE) suite [4]. Pair Distribution Function (PDF) refinements were completed using the software package PDFGUI [5] with a maximum Q value of 30 Å^{-1}. The pair distribution function, $G(r)$, as a func-

Supplementary Table S1: Single crystal refinement parameters for $Yb_2Be_2SiO_7$ at 300 K

Formula weight	504.19 g/mol
Space Group	$P\bar{4}2_1m$
Unit cell	a = 7.207(1) Å
Unit cell	c = 4.719(1) Å
Volume	245.1(2) Å^3
Density (calculated)	6.832 g/cm^3
Extinction coefficient	0.082(3)
Absorption coefficient	38.147 mm^{-1}
F(000)	436
2θ range	8.00 to 66.10°
Total Reflections	4477
Independent reflections	513 [Rint = 0.0550]
Refinement method	Full-matrix least-squares F^2
Absolute structure parameter	0.03(4)
Data / restraints / parameters	559 / 0 / 21
Final R indices	R_1(I 2σ(I)) = 0.0189;
	wR_2 (I 2σ(I)) = 0.0440
	R_1 (all) = 0.0192;
	wR_2 (all) = 0.0442
Largest diff. peak and hole	+1.972 e/Å^{-3}, -1.249 e/Å^{-3}
R.M.S. deviation from mean	0.370 e/Å^{-3}
Goodness-of-fit on F^2	1.149

* hzhou10@utk.edu
† aczelaa@ornl.gov

Supplementary Table S2: Atomic coordinates and isotropic displacement parameters U_{eq} (Å^2) from single-crystal XRD

Atom	Wyck.	x	y	z	U_{eq}
Yb	4e	0.1589(1)	0.6589(1)	0.50772(1)	0.0040(1)
Be	4e	0.637(1)	0.137(1)	0.960(2)	0.006(2)
Si	2a	0	0	0	0.0033(4)
O_1	2c	0	1/2	0.185(2)	0.005(1)
O_2	4e	0.6423(6)	0.1423(6)	0.295(1)	0.005(1)
O_3	8f	0.0824(7)	0.1624(6)	0.203(1)	0.0058(8)

Supplementary Table S3: Fractional coordinates for $Yb_2Be_2SiO_7$ from neutron powder diffraction at 0.25 K

Atom	Wyck.	x	y	z
Yb	4e	0.1590(3)	0.6590(3)	0.506(1)
Be	4e	0.6387(4)	0.1387(4)	0.955(1)
Si	2a	0	0	0
O_1	2c	0	1/2	0.181(2)
O_2	4e	0.6405(6)	0.1405(6)	0.303(1)
O_3	8f	0.0846(5)	0.1658(6)	0.201(1)

tion of distance, r(Å), is shown for the three samples in Supplementary Fig. S1. All three PDF patterns are well-explained by the known crystal structure of these materials with no clear evidence for significant deviations that would arise from local structure distortions. It is also worth noting that the PDF patterns for polycrystalline and crushed single crystal $Yb_2Be_2SiO_7$ are nearly identical.

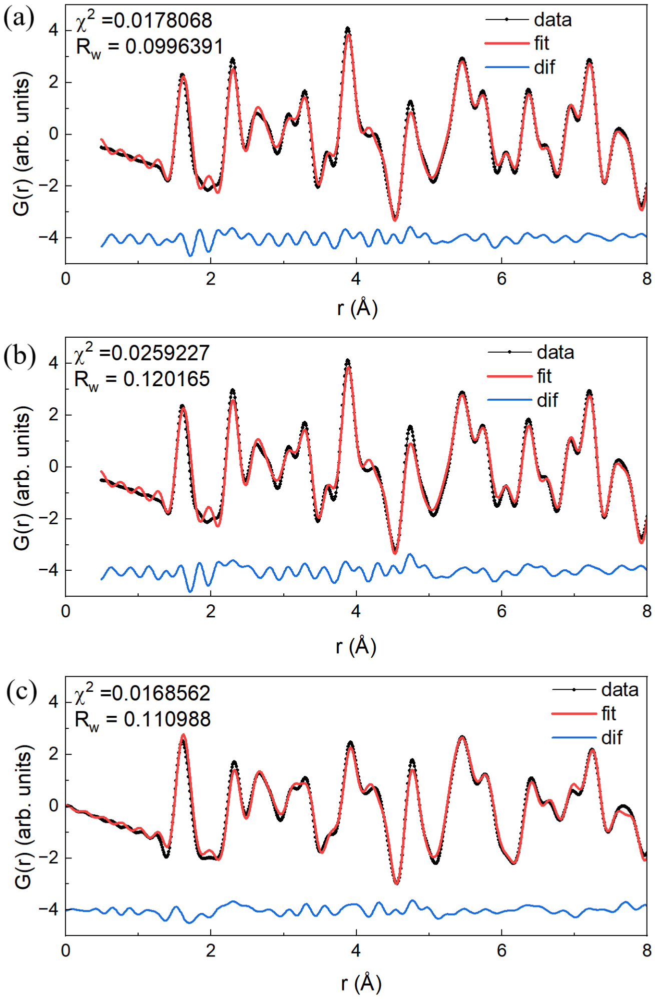

Supplementary Figure S1: **Local structure investigation.** The pair distribution function $G(r)$ vs distance for (a) polycrystalline $Yb_2Be_2SiO_7$, (b) crushed single crystal $Yb_2Be_2SiO_7$, and (c) polycrystalline $Er_2Be_2SiO_7$. The data are described well by the known crystal structures for these materials, so there is no evidence for local structure distortions.

II. HALF-POLARIZED NEUTRON POWDER DIFFRACTION

The magnetic susceptibility χ is a measure of the magnetization of a material in an applied magnetic field. In most crystalline materials, the influence of spin-orbit coupling induces anisotropy that results in χ taking the form of a second-rank tensor rather than a scalar quantity. The components of this tensor can be determined by the half-polarized neutron powder diffraction (pNPD) technique in the linear M/H regime and they provide insight into the local anisotropy of the magnetic ion [6]. The atomic site symmetry is used to establish appropriate constraints for χ. Spin-up and spin-down neutron diffraction patterns with intensities of I^+ and I^- are measured separately, and then Rietveld refinements of the sum and difference patterns (given by $I^+ + I^-$ and I^+ - I^- respectively) are performed using the software CrysPy [7].

The results of these Rietveld refinements for the $Yb_2Be_2SiO_7$ HB-2A data collected at 5 K and 1.5 T are shown in Supplementary Fig. S2. The local site susceptibility tensor extracted from this analysis is given by:

$$\chi_{ij} = \begin{pmatrix} -0.12(3) & -0.02(2) & 0.05(4) \\ -0.02(2) & -0.12(3) & 0.05(4) \\ 0.05(4) & 0.05(4) & 2.36(3) \end{pmatrix}$$

The magnetization ellipsoids' principal axis directions and magnitudes can be obtained from the local site susceptibility tensor. For $Yb_2Be_2SiO_7$, we find that the two principal g-tensor directions not constrained by symmetry are nearly aligned with the crystallographic [001]-axis and the dimer bond direction ([110] and [1$\bar{1}$0] for the two dimer sublattices). The magnitudes of the principal axes are 2.32 μ_B/T, 0.14 μ_B/T, and 0.10 μ_B/T, with the largest value for the pseudo-[001]-axis direction. The magnetization ellipsoids are plotted in Fig. 2(e) of the main manuscript.

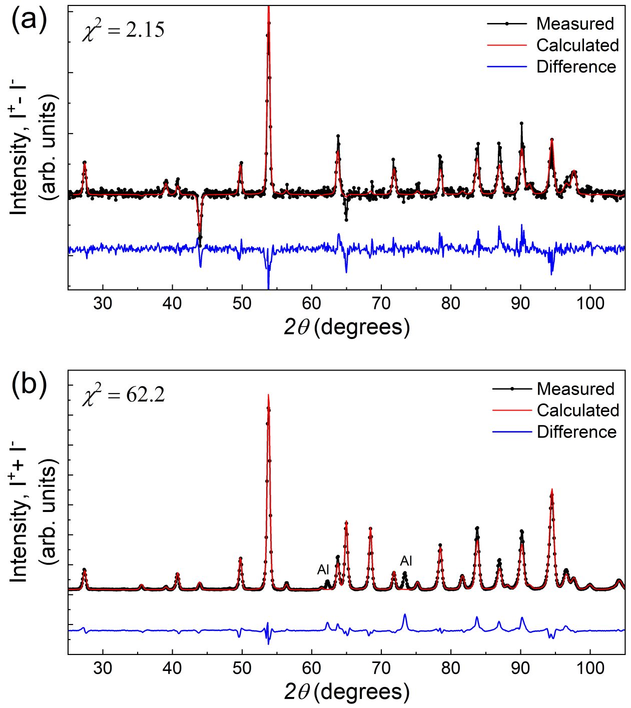

Supplementary Figure S2: **pNPD Rietveld refinement results.** (a) The difference pattern, I^+ - I^-, and (b) the sum pattern, $I^+ + I^-$, obtained from the pNPD measurements at 5 K under an applied field of 1.5 T. For each case, the Rietveld refinement is superimposed on the data and the fit residual is shown below it. The two peaks from the Al sample can are labeled in (b).

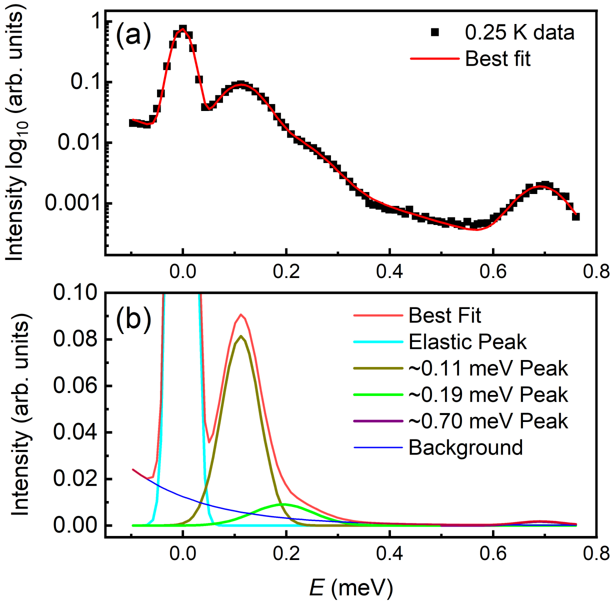

Supplementary Figure S3: **Low-energy neutron spectroscopy peak fitting.** (a) Constant-Q cut of CNCS data (Q-integration range [0.3, 1.8] Å^{-1}) with E_i = 1.55 meV and T = 0.25 K. The best fitting result using a function with four Gaussian peaks, a decaying exponential term, and a constant is superimposed on the data. (b) The contribution of each Gaussian peak to the final fit result. The extracted peak parameters are given in Supplementary Table S4.

Supplementary Table S4: CNCS data fitting results at 0.25 K with E_i = 1.55 meV using a four Gaussian peak model

Peak center	Peak width(FWHM)	Peak Amplitude
-0.0019(2)	0.0390(3)	1
0.111(2)	0.088(4)	0.25(3)
0.19(4)	0.14(5)	0.04(3)
0.692(8)	0.10(2)	0.006(2)

III. ISOLATED DIMER MODEL

For $Yb_2Be_2SiO_7$, the first excited CEF level is well-separated from the ground state which ensures that an effective spin-1/2 model is applicable at sufficiently low-temperatures. We construct the spin operators within a dimer unit as:

$$\begin{aligned} S_m^\alpha &= \frac{1}{2}(\sigma_\alpha \otimes \mathbf{1}) \\ S_n^\beta &= \frac{1}{2}(\mathbf{1} \otimes \sigma_\beta) \end{aligned} \tag{S1}$$

where $\alpha, \beta = x, y, z$, $\mathbf{1}$ represents the identity matrix, $\sigma_{\alpha,\beta}$ denotes the Pauli matrices and $\otimes$ is the Kronecker product.

The strong spin-orbit coupling of the Yb^{3+} ions and the low-symmetry of the crystal structure ensures that a Hamiltonian with XYZ intradimer exchange is required to explain the data. The proposed intradimer Hamiltonian with the associated Zeeman term is shown in Eq. 1 of the main manuscript. Diagonalizing this Hamiltonian generates the following eigenvalues:

$$\begin{aligned} E_0 &= \frac{1}{4}(-J_{xx}^{A/B} + J_{yy}^{A/B} + J_{zz}^{A/B}) \\ E_1 &= \frac{1}{4}(J_{xx}^{A/B} + J_{yy}^{A/B} - J_{zz}^{A/B}) \\ E_2 &= \frac{1}{4}(J_{xx}^{A/B} - J_{yy}^{A/B} + J_{zz}^{A/B}) \\ E_3 &= \frac{1}{4}(-J_{xx}^{A/B} - J_{yy}^{A/B} - J_{zz}^{A/B}) \end{aligned} \tag{S2}$$

We used the zero-field heat capacity and the neutron spectroscopy data to establish the experimental eigenvalues for $Yb_2Be_2SiO_7$. Neutron spectroscopy data collected at CNCS with E_i = 1.55 meV reveals three magnetic excitations. The precise energy and peak width

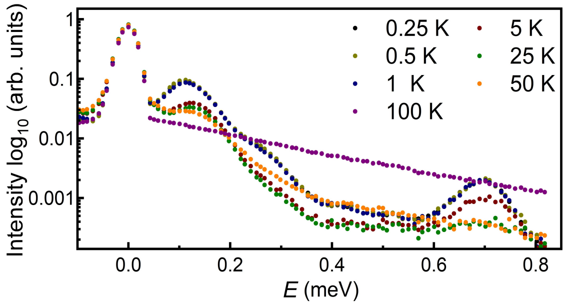

Supplementary Figure S4: ***T*-dependence of low-energy excitations.** (a) Constant-Q cut of CNCS data (Q-integration range [0.3, 1.8] Å^{-1}) with $E_i = 1.55$ meV at several temperatures between 0.25 K and 100 K.

values were extracted from the constant-Q cut shown in Supplementary Fig. S3 using a fitting function consisting of four Gaussian peaks, a decaying exponential term, and a constant. These parameters are provided in Supplementary Table S4. The temperature-dependence of these modes, presented in Supplementary Fig. S4, suggests that they have a magnetic origin. There is also a fourth higher-energy magnetic excitation that was observed in the $E_i = 2.49$ meV data presented in the main manuscript. To assess which excitations could be associated with single dimer physics, we simulated our low-T heat capacity data with the function [8]:

$$C_m(T) = \frac{1}{k_B T^2}\Bigg\{-\left(\frac{1}{Z}\sum_j E_j \ \exp\left(-\frac{E_j}{k_B T}\right)\right)^2 + \frac{1}{Z}\sum_j E_j^2 \ \exp\left(-\frac{E_j}{k_B T}\right)\Bigg\} \quad \text{(S3)}$$

where E_j is the eigenvalue of dimer state j, $Z = \sum_j \exp\left(-\frac{E_j}{k_B T}\right)$ is the partition function, and k_B is the Boltzmann factor. The XYZ Hamiltonian can yield up to three single-dimer excitations, so we tried different eigenvalue combinations in our simulations that were consistent with the four energy levels observed in neutron spectroscopy. Several of these simulation results are presented in Supplementary Fig. S5. We found that the two higher-energy excitations have negligible contributions to the measured heat capacity, so we do not consider them further in our single-dimer models. The best agreement between the data and the simulation is found for a single-dimer model with a doubly-degenerate 0.11 meV excitation and a non-degenerate 0.19 meV excitation.

The XYZ Hamiltonian has 12 solutions that are consistent with this established eigenvalue scheme, so we simulated the neutron spectroscopy, magnetization, magnetic susceptibility, and field-induced heat capacity for each of these models. The neutron spectroscopy simulations of the dynamical structure factor for transitions between

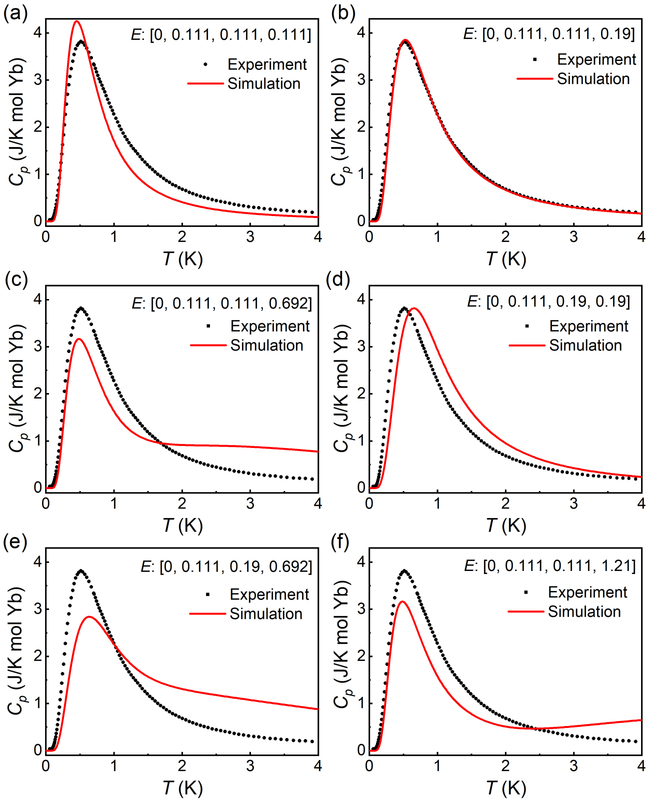

Supplementary Figure S5: **Zero-field heat capacity modeling.** Simulations for different single-dimer models are superimposed on the data in panels (a-f). The simulation with a doubly-degenerate 0.11 meV excitation and a non-degenerate 0.19 meV excitation describes the data best.

the single dimer levels j and k of the XYZ model were performed using the expression [9]:

$$S(\mathbf{Q},E) = A\sum_{j,k}\exp\left(-\frac{E_j}{k_B T}\right)\sum_{\alpha,\beta}\left(\delta_{\alpha\beta} - \frac{Q_\alpha Q_\beta}{Q^2}\right)\times \sum_{m,n} f_n^*(Q) f_m(Q)\ \exp\Big(i\mathbf{Q}\cdot(\vec{R}_m - \vec{R}_n)\Big)\times \langle\psi_j|\,M_m^\alpha\,|\psi_k\rangle \times \langle\psi_k|\,M_n^\beta\,|\psi_j\rangle\,\delta(E + E_j - E_k) \quad \text{(S4)}$$

where $\alpha, \beta = x, y, z$, S_m^α refers to the α component of the effective spin-1/2 operator for magnetic ion m, $g_{\alpha\beta}^m$ represents one component of the g-tensor for magnetic ion m, $\mathbf{R}_m$ and $f_m(Q)$ are the position vector and magnetic form factor for magnetic ion m, ψ_j is the eigenfunction of dimer state j, and $M_m^\alpha = \sum_\gamma g_{\alpha\gamma}^m S_m^\gamma$. The quantity A includes a constant and the Debye-Waller factor $exp(-2W)$. The neutron spectroscopy simulation was powder-averaged to facilitate direct comparison to the CNCS data using the equation:

$$S(Q,E) = \int \frac{d\Omega}{4\pi} S(\mathbf{Q},E) \quad \text{(S5)}$$

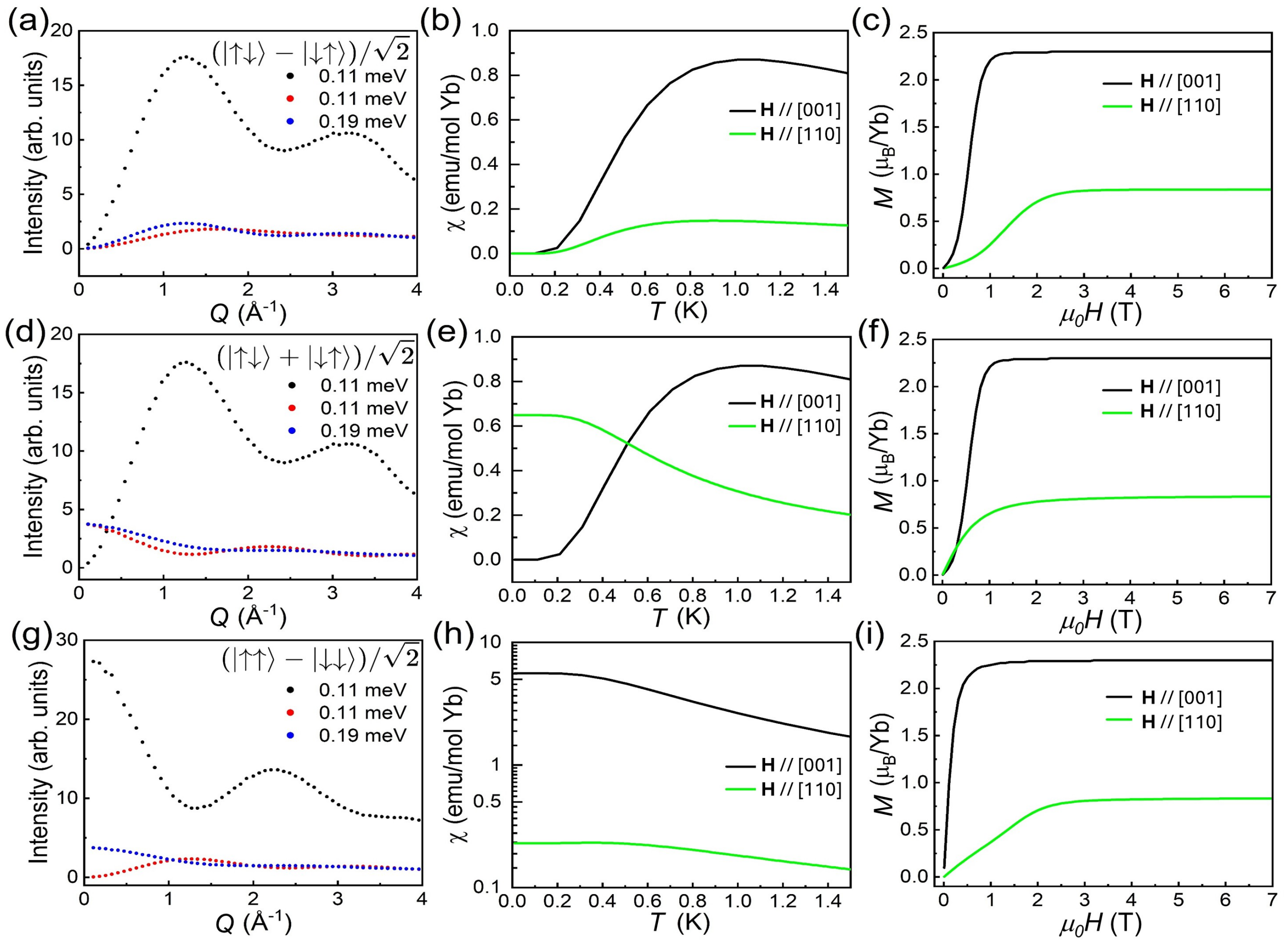

Supplementary Figure S6: **Simulations for XYZ dimer models with different ground states.** (a) Simulated powder dynamical structure factor $S(Q)$ for the three excitations, (b) simulated single crystal magnetic susceptibility as a function of temperature, and (c) simulated single crystal anisotropic magnetization at 0.4 K for an isolated dimer model with anisotropic exchange using the parameters $J_{xx} = 0.03$ meV, $J_{yy} = 0.19$ meV, and $J_{zz} = 0.19$ meV with the dimer ground state $\frac{1}{\sqrt{2}}(|\uparrow\downarrow\rangle - |\downarrow\uparrow\rangle)$. (d-f) Similar simulations for a second isolated dimer model using the parameters $J_{xx} = -0.19$ meV, $J_{yy} = -0.03$ meV, and $J_{zz} = 0.19$ meV with the dimer ground state $\frac{1}{\sqrt{2}}(|\uparrow\downarrow\rangle + |\downarrow\uparrow\rangle)$. (g-i) Similar simulations for a third isolated dimer model using the parameters $J_{xx} = 0.19$ meV, $J_{yy} = -0.03$ meV, and $J_{zz} = -0.19$ meV with the dimer ground state $\frac{1}{\sqrt{2}}(|\uparrow\uparrow\rangle - |\downarrow\downarrow\rangle)$.

The magnetization simulations were performed using the expression:

$$M(\mathbf{H},T) = \frac{1}{N}\sum_m \frac{1}{Z}\sum_j \langle\psi_j(\mathbf{H})|\,\mu_B\,\mathbf{g}_m\cdot\mathbf{S}_m\,|\psi_j(\mathbf{H})\rangle \times \exp\left(-\frac{E_j}{k_B T}\right) \quad \text{(S6)}$$

where N is the number of magnetic ions in a unit cell, $\psi_j(\mathbf{H})$ is the eigenfunction of dimer state j in an applied magnetic field $\mathbf{H}$, $\mathbf{g}_m$ is the g-tensor of magnetic ion m, and $\mathbf{S}_m$ is the effective spin-1/2 operator of magnetic ion m. The DC susceptibility was simulated by taking derivatives of the magnetization as:

$$\chi(\mathbf{H},T) = \frac{\partial M(\mathbf{H},T)}{\partial H}. \quad \text{(S7)}$$

Within a linear response regime of small $\mathbf{H}$, $\chi(\mathbf{H},T)$ remains a constant. The field-dependent heat capacity simulations were performed using Supplementary Eq. S3 with modified eigenvalues obtained by diagonalizing the XYZ Hamiltonian with the appropriate Zeeman term included.

Supplementary Fig. S6 presents simulations of the powder-averaged dynamical structure factors, the low-T magnetic susceptibility, and the $\mathbf{H}\parallel[001]$ and [110]

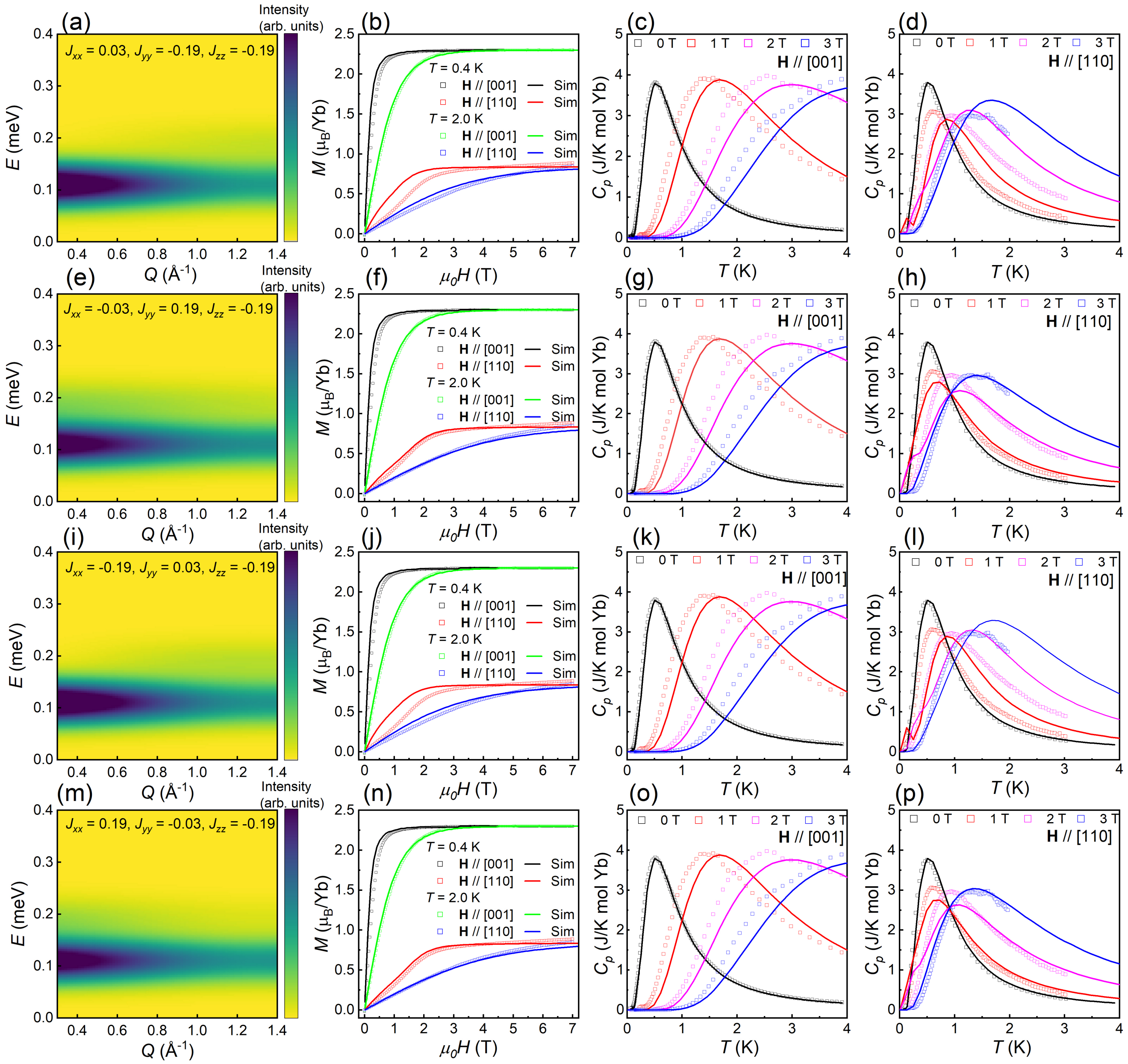

Supplementary Figure S7: **Detailed comparison of the four most probable XYZ dimer models for $\mathbf{Yb_2Be_2SiO_7}$.** (a) Simulated dynamical structure factor $S(Q,E)$ for the three excitations, (b) simulated and measured anisotropic magnetization at both 0.4 K and 2 K, (c) simulated and measured heat capacity data for $\mathbf{H} \parallel [001]$, and (d) simulated and measured heat capacity data for $\mathbf{H} \parallel [110]$. The simulations correspond to an isolated dimer model with anisotropic exchange using the parameters $J_{xx} = 0.03$ meV, $J_{yy} = -0.19$ meV, and $J_{zz} = -0.19$ meV. Similar dynamical structure factor simulations and the same bulk characterization data with similar simulations superimposed on it for (e-h) an isolated dimer model with $J_{xx} = -0.03$ meV, $J_{yy} = 0.19$ meV, and $J_{zz} = -0.19$ meV, (i-l) an isolated dimer model with $J_{xx} = -0.19$ meV, $J_{yy} = 0.03$ meV, and $J_{zz} = -0.19$ meV, and (m-p) an isolated dimer model with $J_{xx} = 0.19$ meV, $J_{yy} = -0.03$ meV, and $J_{zz} = -0.19$ meV. The second and fourth set of simulations show the best agreement with the experimental data.

magnetization for representative XYZ dimer models with the known g-tensor and eigenvalues for $Yb_2Be_2SiO_7$ and $\frac{1}{\sqrt{2}}(|\uparrow\downarrow\rangle - |\downarrow\uparrow\rangle)$, $\frac{1}{\sqrt{2}}(|\uparrow\downarrow\rangle + |\downarrow\uparrow\rangle)$, and $\frac{1}{\sqrt{2}}(|\uparrow\uparrow\rangle - |\downarrow\downarrow\rangle)$ ground states. In the regime $Q \leq 1.6$ Å^{-1}, the $S_z = 0$ models have intense modes with a dynamical structure factor,

$$S(Q) = A\left(1 - \frac{sin(Qd)}{Qd}\right) \tag{S8}$$

where A is a constant and d is the intradimer distance. One can distinguish between them by measuring the Q-dependence of the weaker mode intensities, the low-T magnetic susceptibility, or the low-T anisotropic magnetization. All models with $\frac{1}{\sqrt{2}}(|\uparrow\downarrow\rangle - |\downarrow\uparrow\rangle)$ ground states have low-field magnetization plateaus and sharp drops in the magnetic susceptibility with decreasing T when the magnetic field is applied both parallel and perpendicular to the quantization axis, while the models with $\frac{1}{\sqrt{2}}(|\uparrow\downarrow\rangle + |\downarrow\uparrow\rangle)$ ground states only have low-field magnetization plateaus and sharp drops in the magnetic susceptibility with decreasing T when the magnetic field is applied along the quantization axis. All models with $\frac{1}{\sqrt{2}}(|\uparrow\uparrow\rangle + |\downarrow\downarrow\rangle)$ and $\frac{1}{\sqrt{2}}(|\uparrow\uparrow\rangle - |\downarrow\downarrow\rangle)$ ground states have intense modes with a dynamical structure factor well-described by the function

$$S(Q) = A\frac{sin(Qd)}{Qd} \tag{S9}$$

in the regime $Q \leq 1.6$ Å^{-1} and exhibit no sharp drops in the susceptibility with decreasing T or low-field magnetization plateaus regardless of the applied field direction.

We identified four models out of the 12 possibilities that were consistent with the Q-dependence and energy of the most intense mode measured by neutron spectroscopy. The eigenvectors for these four models are as follows:

$$\begin{aligned}
|\psi_0\rangle &= \frac{1}{\sqrt{2}}(|\uparrow\uparrow\rangle - |\downarrow\downarrow\rangle) \\
|\psi_1\rangle &= \frac{1}{\sqrt{2}}(|\uparrow\downarrow\rangle + |\downarrow\uparrow\rangle) \\
|\psi_2\rangle &= \frac{1}{\sqrt{2}}(|\uparrow\uparrow\rangle + |\downarrow\downarrow\rangle) \\
|\psi_3\rangle &= \frac{1}{\sqrt{2}}(|\uparrow\downarrow\rangle - |\downarrow\uparrow\rangle)
\end{aligned} \tag{S10}$$

The simulation results of the four models are presented in Supplementary Fig. S7.

The dimer ground state for all four models is characterized by $S_z \neq 0$ wavefunctions, which are stabilized by the large ferromagnetic $J_{zz} = -0.19$ meV that is common to all of them. The four models only produce two different sets of bulk characterization simulations, as exchanging J_{xx} and J_{yy} only affects the Q-dependent intensity of the weak modes measured by neutron spectroscopy. One set of models produces simulations that show much better agreement with the bulk characterization data. Although the Q-dependence of the weak mode intensities is not exactly the same for these two models, our neutron powder spectroscopy data are not sufficient for differentiating between them conclusively. The exchange parameters for one of these models are $J_{xx} = \ 0.19$ meV, $J_{yy} = -0.03$ meV, and $J_{zz} = -0.19$ meV and it has an entangled dimer ground state of $\frac{1}{\sqrt{2}}(|\uparrow\uparrow\rangle - |\downarrow\downarrow\rangle)$. The second model has exchange parameters of $J_{xx} = \ -0.03$ meV, $J_{yy} = 0.19$ meV, and $J_{zz} = -0.19$ meV and it has an entangled dimer ground state of $\frac{1}{\sqrt{2}}(|\uparrow\uparrow\rangle + |\downarrow\downarrow\rangle)$.

[1] Rodríguez-Carvajal, J. Recent advances in magnetic structure determination by neutron powder diffraction. *Physica B: Condensed Matter* **192**, 55–69 (1993).

[2] Kuz'micheva, G. M. *et al.* Preparation, structure, and properties of new laser crystals $Y_2SiBe_2O_7$ and $Y_2Al(BeB)O_7$. *Inorganic Materials* **38**, 60–65 (2002).

[3] Brassington, A., Huang, Q., Aczel, A. A. & Zhou, H. D. Synthesis and magnetic properties of the Shastry-Sutherland family $R_2Be_2SiO_7$ (R = Nd, Sm, Gd-Yb). *Phys. Rev. Mater.* **8**, 014005 (2024).

[4] Mcdonnell, M. *et al.* ADDIE: ADvanced DIffraction Environment – a software environment for analyzing neutron diffraction data. *Acta Crystallographica Section A Foundations and Advances* **73**, a377 (2017).

[5] Farrow, C. L. *et al.* PDFfit2 and PDFgui: Computer programs for studying nanostructure in crystals. *Journal of Physics: Condensed Matter* **19**, 335219 (2007).

[6] Baral, R. *et al.* Magnetic pair distribution function and half polarized neutron powder diffraction at the HB-2A powder diffractometer. *Journal of Magnetism and Magnetic Materials* **630**, 173342 (2025).

[7] Kibalin, I. A. & Gukasov, A. Local magnetic anisotropy by polarized neutron powder diffraction: Application of magnetically induced preferred crystallite orientation. *Phys. Rev. Res.* **1**, 033100 (2019).

[8] Probert, M. Thermodynamic properties of solids: Experiment and modeling. *Contemporary Physics* **53**, 62–63 (2012).

[9] Andres, H. *et al.* Magnetic excitations in polyoxometalate clusters observed by inelastic neutron scattering: Evidence for anisotropic ferromagnetic exchange interactions in the tetrameric cobalt(II) cluster $[Co_4(H_2O)_2(PW_9O_{34})_2]^{10-}$. Comparison with the magnetic and specific heat properties. *Journal of the American Chemical Society* **121**, 10028–10034 (1999).
